# A mild increase in nutrient signaling to mTORC1 in mice leads to parenchymal damage, myeloid inflammation and shortened lifespan

**DOI:** 10.1038/s43587-024-00635-x

**Published:** 2024-06-07

**Authors:** Ana Ortega-Molina, Cristina Lebrero-Fernández, Alba Sanz, Miguel Calvo-Rubio, Nerea Deleyto-Seldas, Lucía de Prado-Rivas, Ana Belén Plata-Gómez, Elena Fernández-Florido, Patricia González-García, Yurena Vivas-García, Elena Sánchez García, Osvaldo Graña-Castro, Nathan L. Price, Alejandra Aroca-Crevillén, Eduardo Caleiras, Daniel Monleón, Consuelo Borrás, María Casanova-Acebes, Rafael de Cabo, Alejo Efeyan

**Affiliations:** 1 Metabolism and Cell Signaling Laboratory, Spanish National Cancer Research Centre (CNIO), Madrid, Spain; 2 Metabolism in cancer and aging Laboratory, Immune System Development And Function Department, Centro de Biología Molecular Severo Ochoa (CBM), Madrid, Spain; 3 Translational Gerontology Branch, National Institute on Aging (NIA), National Institutes of Health (NIH), Baltimore, MD USA; 4 Histopathology Unit, Spanish National Cancer Research Centre (CNIO), Madrid, Spain; 5 Bioinformatics Unit, Spanish National Cancer Research Centre (CNIO), Madrid, Spain; 6 Institute of Applied Molecular Medicine (IMMA-Nemesio Díez), Department of Basic Medical Sciences, School of Medicine, San Pablo-CEU University, CEU Universities, Boadilla del Monte, Madrid, Spain; 7 Cardiovascular Regeneration Program, Centro Nacional de Investigaciones Cardiovasculares, Madrid, Spain; 8 Department of Pathology, University of Valencia, Valencia, Spain; Centro de Investigación Biomédica en Red Fragilidad y Envejecimiento Saludable-Instituto de Salud Carlos III (CIBERFES-ISCIII), Institute of Health Research-INCLIVA, Valencia, Spain; 9 Freshage Research Group, Department of Physiology, Faculty of Medicine, University of Valencia, Centro de Investigación Biomédica en Red Fragilidad y Envejecimiento Saludable-Instituto de Salud Carlos III (CIBERFES-ISCIII), MiniAging Research Group, Institute of Health Research-INCLIVA, Valencia, Spain; 10 Cancer Immunity Laboratory, Spanish National Cancer Research Centre (CNIO), Madrid, Spain

**Keywords:** Nutrient signalling, Experimental models of disease, Inflammation

## Abstract

The mechanistic target of rapamycin complex 1 controls cellular anabolism in response to growth factor signaling and to nutrient sufficiency signaled through the Rag GTPases. Inhibition of mTOR reproducibly extends longevity across eukaryotes. Here we report that mice that endogenously express active mutant variants of RagC exhibit multiple features of parenchymal damage that include senescence, expression of inflammatory molecules, increased myeloid inflammation with extensive features of inflammaging and a ~30% reduction in lifespan. Through bone marrow transplantation experiments, we show that myeloid cells are abnormally activated by signals emanating from dysfunctional RagC-mutant parenchyma, causing neutrophil extravasation that inflicts additional inflammatory damage. Therapeutic suppression of myeloid inflammation in aged RagC-mutant mice attenuates parenchymal damage and extends survival. Together, our findings link mildly increased nutrient signaling to limited lifespan in mammals, and support a two-component process of parenchymal damage and myeloid inflammation that together precipitate a time-dependent organ deterioration that limits longevity.

## Abstract (web summary)

Ortega-Molina et al. demonstrate that mouse models with mild, genetic overactivation of mTOR signaling develop chronic myeloid inflammation, causing reduced healthspan and lifespan, without an increase in tumor incidence.

## Main

The relentless increase in the aged population worldwide imposes medical, socioeconomical and philosophical challenges to mankind. By 2050, the global population aged 65 years or older would have tripled in a century, from 5% to 17% (around 2 billion people)^1^. While the prevention and management of aging-related disorders will improve, the overall comorbidities that occur with the multi-organ physiological decline of older adults impose a challenge unlikely to be controlled by the treatment of single symptoms and disorders. Instead, to intervene in the systemic frailty and health decline associated with aging, we need to understand the processes that precipitate cellular and organ malfunctioning with age. Genetic and pharmacological approaches in model organisms have taught us that the process of aging can be modulated and longevity can be shortened or extended by actioning on the functions of a discrete number of proteins^2,3^. Among these is inhibition of the mechanistic target of rapamycin (mTOR) complex 1 (mTORC1), a master regulator of cellular anabolism in response to cellular nutrients and growth factors^4,5^. Intracellular levels of amino acids, intermediate metabolites of glycolysis, certain lipids and other molecules have dedicated sensors that signal to the Rag GTPases upstream of mTORC1 (refs. ^4,6^). Under nutrient sufficiency, RagC loads GDP, whereas its obligate heterodimeric partner RagA is GTP-loaded. This nucleotide configuration allows the recruitment of mTORC1 to the outer lysosomal surface, where mTORC1 binds the Ras homolog enriched in brain (Rheb) that leads to kinase activation of mTORC1 in a growth-factor-dependent manner^7,8^.

Pharmacological and genetic inhibition of TOR extends longevity in yeast^9^, worms^10,11^ and flies^12,13^. In mice, rapamycin^14,15^ and hypomorphic mTORC1 signaling achieved by genetic means also extends lifespan^16,17^. Conversely, genetic activation of mTORC1 signaling has been linked to shortened longevity in lower organisms, including yeast and worms, but mouse models of increased mTORC1 activity, such as heterozygous or homozygous deletion of *Tsc1* or *Tsc2* (refs. ^18–20^), *Pten*^21–23^ or genetic activation of positive regulators of mTORC1, such as *Akt*, *Pi3k*, *Rheb* or *Rraga*^24–28^, have been invariably incompatible with aging studies due to the early development of specific deleterious pathologies that precluded the analyses of mechanisms and processes underlying organismal aging in mammals.

We have previously engineered two knock-in mice expressing active, GDP-like mutant variants of RagC (also known as *Rragc*)^29^, originally identified in human B cell lymphomas^30–32^. Heterozygous *Rragc*^S74C^ or *Rragc*^T89N^ mice show accelerated development of lymphomas when bred to a lymphoma-prone strain, and a moderate increase in nutrient signaling to mTORC1. Together with a newly generated knock-in strain of mice, expressing a canonical GDP-like mutation (S74N), we now show that in the absence of cooperating oncogenes, a mild increase in nutrient signaling to mTORC1 does not lead to spontaneous tumorigenesis, and instead, these mice show a shortened lifespan. This genetic system allows for the interrogation of the mechanisms underscoring the connections between the nutrient–mTORC1 axis and longevity. Increased nutrient signaling to mTORC1 leads to autonomous organ damage and prominent myeloid inflammation in response to evoking signals from damaged organs. Late-life blockade of myeloid cell infiltration mitigates several of these phenotypes and extends lifespan.

## Results

### *Rragc*-mutant mice have shortened lifespan

**Fig. 1:**
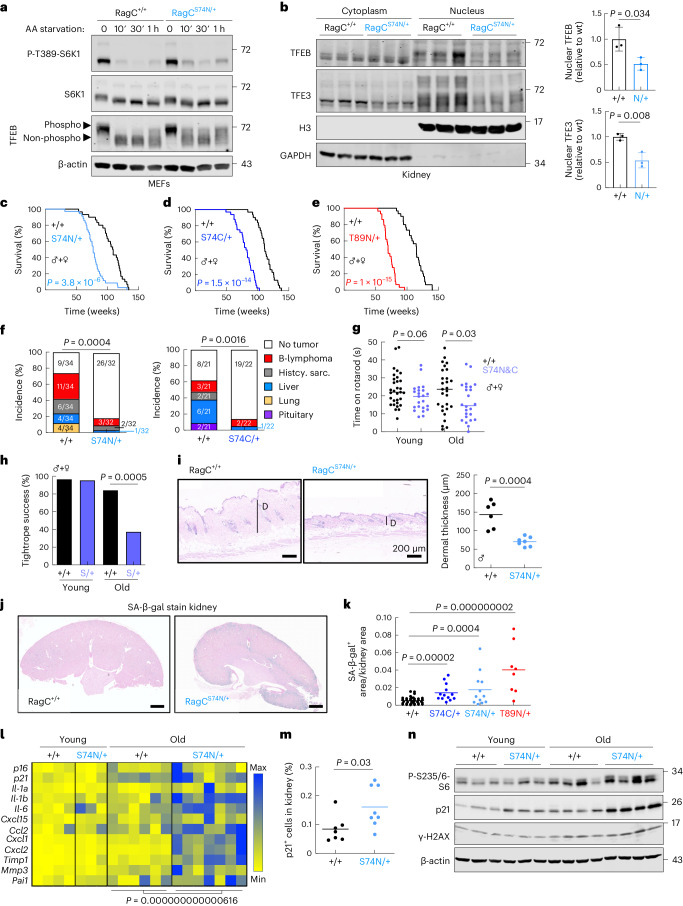
*Rragc*-mutant mice have a shortened lifespan. **a***, Rragc*^+/+^ and *Rragc*^S74N/+^ MEFs were deprived of all amino acids in RPMI with dialyzed serum for 10 min, 30 min and 1 h. Whole-cell protein lysates were immunoblotted for the indicated proteins. **b**, Tfe3 and Tfeb protein levels in subcellular fractions from kidneys from young *Rragc*^+/+^ (*n* = 3) and *Rragc*^S74N/+^ (*n* = 3) mice fasted 16 h. Quantification of Tfe3 and Tfeb levels in the nucleus is relative to histone 3 levels. Data are presented as mean ± s.d. **c**–**e**, Kaplan–Meier survival curves of *Rragc*^+/+^ (*n* = 30) and *Rragc*^S74N/+^ (*n* = 35) (**c**); *Rragc*^+/+^ (*n* = 31) and *Rragc*^S74C/+^ (*n* = 32) (**d**); *Rragc*^+/+^ (*n* = 30) and *Rragc*^T89N/+^ (*n* = 29) (**e**) mice. **f**, Tumor incidence in cohorts from *Rragc*^+/+^ (*n* = 34) and *Rragc*^S74N/+^ (*n* = 32) (left) and from *Rragc*^+/+^ (*n* = 21) and *Rragc*^S74C/+^ (*n* = 22) (right). Percentage of mice with no tumors, B cell lymphomas, histiocytic sarcomas, hepatocellular carcinomas, lung carcinomas and pituitary tumors. **g**, Time on rotarod measured in young (4–7.5-mo-old) and old (14.5–20-mo-old) *Rragc*^+/+^ (young, *n* = 31; old, *n* = 32) and *Rragc*^S74N/+^ and *Rragc*^S74C/+^ (young, *n* = 23; old, *n* = 24) mice. **h**, Tightrope assay performed in the same groups of mice as in **g**. Scale bars represent the percentage of mice that passed the assay. **i**, Dermal thickness measured in back skin of 18-mo-old *Rragc*^+/+^ (*n* = 6) and *Rragc*^*S74N*/+^ (*n* = 7) males. Scale bars, 200 μm. **j**, Representative pictures of SA-β-gal^+^ in kidney. Scale bars, 1,000 μm. **k**, Quantification of SA-β-gal^+^ within the kidney of 18-mo-old *Rragc*^+/+^ (*n* = 38), *Rragc*^S74*C*/+^ (*n* = 13), *Rragc*^S74N/+^ (*n* = 12) and *Rragc*^T89N/+^ (*n* = 8) mice. **l**, qRT–PCR analysis of genes of the SASP in kidneys of young (4-mo-old) and old (18-mo-old) *Rragc*^+/+^ (young, *n* = 4; old, *n* = 6) and *Rragc*^S74N/+^ (young, *n* = 3; old, *n* = 7) male mice. **m**, Quantification of IHC staining of anti-p21 in kidneys collected from 18-mo-old *Rragc*^+/+^ (*n* = 7) and *Rragc*^S74N/+^ (*n* = 8) male mice. **n**, Immunoblot for the indicated proteins from 4-mo-old and 18-mo-old *Rragc*^+/+^ and *Rragc*^*S74N*/+^ male mice. Statistical significance was assessed by log-rank test (**c**–**e**); two-sided chi-squared test (**f**); two-sided Fisher’s exact test (**h**); two-tailed Student’s *t*-test (**b**,**g**,**i**,**k**,**m**); and two-way analysis of variance (ANOVA) (**l**). mo, month. Source data

**Extended Data Fig. 1:**
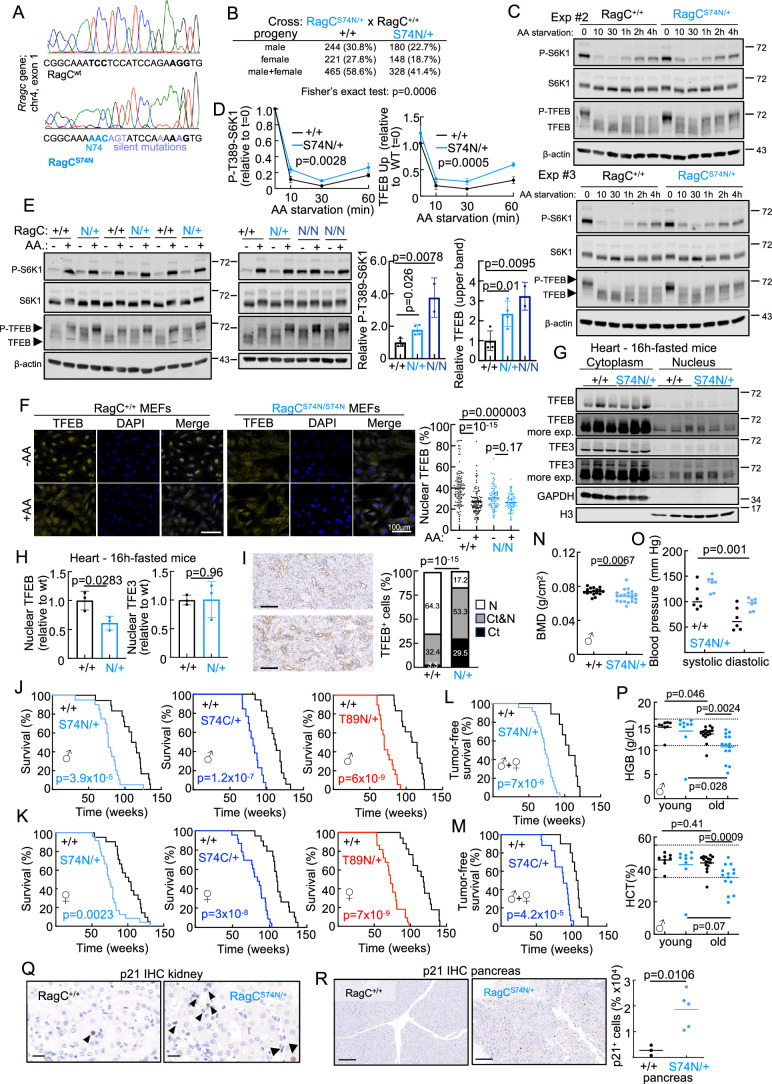
Rragc-mutant mice have shortened lifespan. **a**. Chromatogram of the *Rragc*^wt^ and *Rragc*^S74N^ allele. *Rragc*^S74N^ allele encodes two amino acid substitutions (blue bold), plus silent diagnostic mutations (purple). **b**. Progenies of *Rragc*^S74N^ breeding schemes. **c**. MEFs from *Rragc*^+/+^ and *Rragc*^S74N/+^ mice were deprived of all amino acids in RPMI medium with dialyzed serum for the indicated time-points. **d**. Quantification of P-T389-S6K1 and TFEB upper band (phosphorylated) from triplicates from Fig. 1a and Figure S1c (Statistical significance was calculated by two-tailed Student’s t-test for AUC). Data are presented as mean values ± SD. **e**. (Left) MEFs from *Rragc*^+/+^ (n = 4 biologically independent cells), *Rragc*^S74N/+^ (n = 4 biologically independent cells), and *Rragc*^S74N/S74N^ (n = 2 biologically independent cells), mice were deprived from and re-stimulated with amino acids for 10 min. (Right) Quantification of P-T389-S6K1 and TFEB upper band in -AA samples. Data are presented as mean values ± SD **f**. (Left) Immunofluorescence of TFEB in *Rragc*^+/+^ and Rragc^S74N/S74N^ MEFs in RPMI with and without amino acids; (Right) with the percentage of nuclear TFEB per cell (*Rragc*^+/+^ + AA n = 112 –AA n = 117; *Rragc*^S74N/S74N^ + AA n = 72 –AA n = 91). Bars represent 100μm **g**. TFE3 and TFEB levels fractions of hearts from *Rragc*^+/+^ (n = 3) and *Rragc*^S74N/+^ (n = 3) mice after fasting. **h**. Quantification of TFE3 and TFEB levels in the nucleus. Data are presented as mean values ± SD. **i**. Representative anti-TFEB IHC (left) and quantification (right) of TFEB-positive cells in the cytoplasm (Ct), nucleus (N) and cytoplasm&nucleus (Ct&N) in renal tubules from young *Rragc*^+/+^ (n = 4) and *Rragc*^S74N/+^ (n = 4) male mice. Scale bar 100 µm. **j**. Kaplan–Meier survival curves of *Rragc*^+/+^ (n = 18) and *Rragc*^S74N/+^ (n = 20) male mice (left), *Rragc*^+/+^ (n = 19) and *Rragc*^S74C/+^ (n = 13) male mice (center) and *Rragc*^+/+^ (n = 18) and *Rragc*^T89N/+^ (n = 19) male mice (right). **k**. Kaplan–Meier survival curves of *Rragc*^+/+^ (n = 21) and *Rragc*^S74N/+^ (n = 24) female mice (left), *Rragc*^+/+^ (n = 19) and *Rragc*^S74C/+^ (n = 23) female mice (center) and *Rragc*^+/+^ (n = 17) and *Rragc*^T89N/+^ (n = 22) female mice (right). **l**. Kaplan–Meier survival curves of *Rragc*^+/+^ (n = 9) and *Rragc*^S74N/+^ (n = 24) mice that were free of detectable malignant tumors at the time of death. **m**. Kaplan–Meier survival curves of *Rragc*^+/+^ (n = 10) and *Rragc* ^S74C/+^ (n = 17) mice without malignant tumors. In j-m, statistical significance was calculated with the log-rank test. **n**. Bone mineral density (BMD) measured in 13.5-21.5-month-old *Rragc*^+/+^ (n = 17) and *Rragc*^S74N/+^ (n = 21) male mice. **o**. Blood pressure in 16-19-month-old *Rragc*^+/+^ (n = 6) and *Rragc*^S74N/+^ (n = 7) male mice. **p**. Hematological parameters in 3–5- and 18-month-old *Rragc*^+/+^ (young, n = 8; old, n = 16) and *Rragc*^S74N/+^ (young, n = 9; old, n = 14) male mice. **q**. Representative anti-p21 IHC in kidneys from 18-month-old *Rragc*^+/+^ and *Rragc*^S74N/+^ male mice. Bars represents 20 μm. Arrows indicate p21-positive cells. **r**. Representative anti-p21 IHC (left) and quantification (right) from pancreas harvested from 18-month-old *Rragc*^+/+^ (n = 3) and *Rragc*^S74N/+^ (n = 5) male mice. Bars represent 200 μm. Statistical significance in e, h, n, p and r was calculated by two-tailed Student’s t-test. Statistical significance in f was calculated by one-way ANOVA and Tukey’s multiple comparisons test. Statistical significance in a and i was calculated by by two-sided Fisher’s exact test. Statistical significance in o was calculated by Two-way Anova. Source data

We have previously reported knock-in mice expressing activating mutant variants of *Rragc* (*Rragc*^S74C^ and *Rragc*^T89N^)^29^. These mutations encode amino acid substitutions translating from single-nucleotide changes recurrently found in human B cell lymphomas at the equivalent positions S75 and T90 in human *RRAGC*^30^. We have now knocked in a ‘canonical’ GDP-bound, activating RagC mutation, translating into *Rragc*^S74N^ (refs. ^33,34^) and not found in human lymphomas as two contiguous genetic mutations must occur. We engineered a two-nucleotide substitution in *Rragc* exon 1, which translates into the serine-to-asparagine change in aminoacidic position 74, together with additional silent mutations (Extended Data Fig. 1a). Heterozygous *Rragc*^S74N/+^ mice, as seen in *Rragc*^S74C/+^ and *Rragc*^T89N/+^ mice were found at sub-Mendelian ratios (Extended Data Fig. 1b). As previously observed in *Rragc*^S74C/+^ and *Rragc*^T89N/+^ cells^29^, primary mouse embryonic fibroblasts (MEFs) from *Rragc*^S74N/+^ mice show a modest increase in mTORC1 activity, as seen by the phosphorylation status of the mTORC1 targets S6k1 and Tfeb (Fig. 1a and Extended Data Fig. 1c–e), indicating that the effects of these three activating mutations in RagC on mTORC1 signaling are very similar. *Rragc*^S74N/S74N^ mice are not viable^28,29^, but *Rragc*^S74N/S74N^ MEFs show an increase in phospho-S6K1, plus a remarkable upshift of Tfeb, indicative of a large increase in its phosphorylation (Extended Data Fig. 1e). Immunofluorescence-based staining of Tfeb showed the expected nucleo-cytoplasmic shuttle of Tfeb upon amino acid-starvation/replenishment of *Rragc*^+/+^ MEFs, but cytoplasmic retention of Tfeb in *Rragc*^S74N/S74N^ MEFs even under amino acid starvation (Extended Data Fig. 1f). Tissues from heterozygous *Rragc*^S74N/+^ fasted mice present decreased Tfeb and Tfe3 in the nucleus (Fig. 1b and Extended Data Fig. 1g,h). Increased cytoplasmic retention of Tfeb in *Rragc*^S74N/+^ mice was validated by quantification of immunohistochemical stain of Tfeb in kidneys (Extended Data Fig. 1i).

When bred to the follicular lymphoma-prone strain *VavP-Bcl2*^Tg^^35^, *Rragc*^mut/+^ mice rapidly develop lymphomas^29^, but in the absence of additional genetic modifications, the expression of *Rragc*^mut^ in heterozygosity lead to a strikingly similar shortened lifespan in all three *Rragc*^mut/+^ male and female cohorts (Fig. 1c–e and Extended Data Fig. 1j,k).

We first reasoned that the premature death of *Rragc*^mut/+^ mice was a consequence of spontaneous tumor development; however, and surprisingly for full-body expression of bona fide oncogenic mutations, upon postmortem histopathological examination, *Rragc*^S74N/+^ and *Rragc*^S74C/+^mice showed reduced, rather than increased, spontaneous tumor incidence (Fig. 1f). When all sacrificed mice with tumors identified post hoc were removed from the Kaplan–Meier survival curves, tumor-free *Rragc*^S74N/+^ and *Rragc*^S74C/+^ mice still showed a shortened lifespan compared to tumor-free *Rragc*^+/+^ mice (Extended Data Fig. 1l, m), indicating that the premature death of *Rragc*^mut/+^ mice is not related to differences in spontaneous tumorigenesis. Instead, we found multiple canonical markers of aging^36^ in ~16-month-old *Rragc*^mut/+^ mice: loss of neuromuscular coordination in rotarod and tightrope tests (Fig. 1g,h), thinning of the dermal layer of the skin (Fig. 1i), loss of bone mineral density (Extended Data Fig. 1n), increased systolic and diastolic pressure (Extended Data Fig. 1o) and decreased hemoglobin and hematocrit (Extended Data Fig. 1p). We also quantified senescence-associated β-galactosidase (SA-β-gal) activity, a readout of cellular senescence (a process strongly associated with aging^37^), in organs from ~16-month-old wild-type (wt) and *Rragc*^mut/+^ mice (Fig. 1j). Automatic quantification of SA-β-gal revealed a significant increase in the kidneys from the three *Rragc*^mut/+^ strains (Fig. 1k). Consistently, the expression of markers of senescence and a senescence-associated secretory phenotype (SASP) was also increased (Fig. 1l). In addition, immunohistochemistry (IHC) staining for Cdkn1a (p21) revealed significantly increased abundance of p21-positive cells in the kidney and pancreas from *Rragc*^S74N/+^ mice (Fig. 1m and Extended Data Fig. 1q,r), a difference confirmed by immunoblot (Fig. 1n). Additional markers of damage associated with aging, such as phosphorylation of H2ax (γ-H2ax; Fig. 1n), were also augmented in *Rragc*^S74N/+^ samples compared to *Rragc*^+/+^ samples of an identical chronological age.

### Old *Rragc*-mutant mice show an inflammaging phenotype

**Fig. 2:**
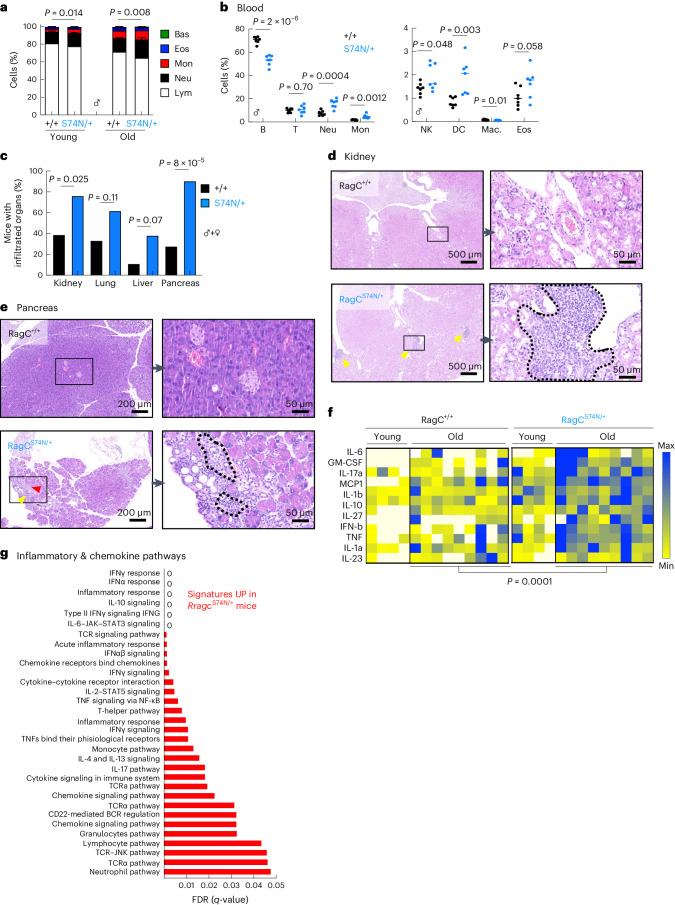
Old *Rragc*-mutant mice show an inflammaging phenotype. **a**, White blood cell (WBC) count performed in young (3–5-mo-old) and old (18-mo-old) *Rragc*^+/+^ (young, *n* = 8; old, *n* = 9) and *Rragc*^*S74N*/+^ (young, *n* = 12; old, *n* = 12) males. Each colored stack represents cell-type percentage (Lym, lymphocyte; Neu, neutrophil; Mon, monocyte; Eos, eosinophil; Bas, basophil). Data are presented as mean ± s.d. **b**, Percentage of the indicated cell populations in the blood of 18-mo-old *Rragc*^+/+^ (*n* = 9) and *Rragc*^S74N/+^ (*n* = 9) male mice. B, B cell; T, T cell; NK, natural killer; DC, dendritic cell; Mac, macrophage. **c**, Incidence of infiltrated inflammatory cells in the indicated tissues from 18-mo-old *Rragc*^+/+^ (*n* = 18) and *Rragc*^S74N/+^ (*n* = 21) mice. **d**, Representative H&E pictures in the same mice as in **c**, showing inflammatory foci in kidney (dashed lines). **e**, Representative H&E pictures in the same mice as in **c**, showing inflammatory foci in pancreas (dashed lines). **f**, Quantification of inflammatory cytokines in sera from young (4-mo-old) and old (18-mo-old) *Rragc*^+/+^ (young, *n* = 4; old, *n* = 9) and *Rragc*^S74N/+^ (young, *n* = 4; old, *n* = 9) male mice measured by Legendplex assay using flow cytometry. **g**, Graphical representation of the false discovery rates (FDRs) from the indicated KEGG, Hallmark, REACTOME and WikiPathways gene sets enriched in kidneys from 18-mo-old *Rragc*^S74N/+^ (*n* = 5) versus *Rragc*^+/+^ (*n* = 5) mice. Statistical significance was calculated by two-way ANOVA (**a**,**f**); two-tailed Student’s *t*-test (**b**); and two-sided Fisher’s exact test (**c**). Source data

**Extended Data Fig. 2:**
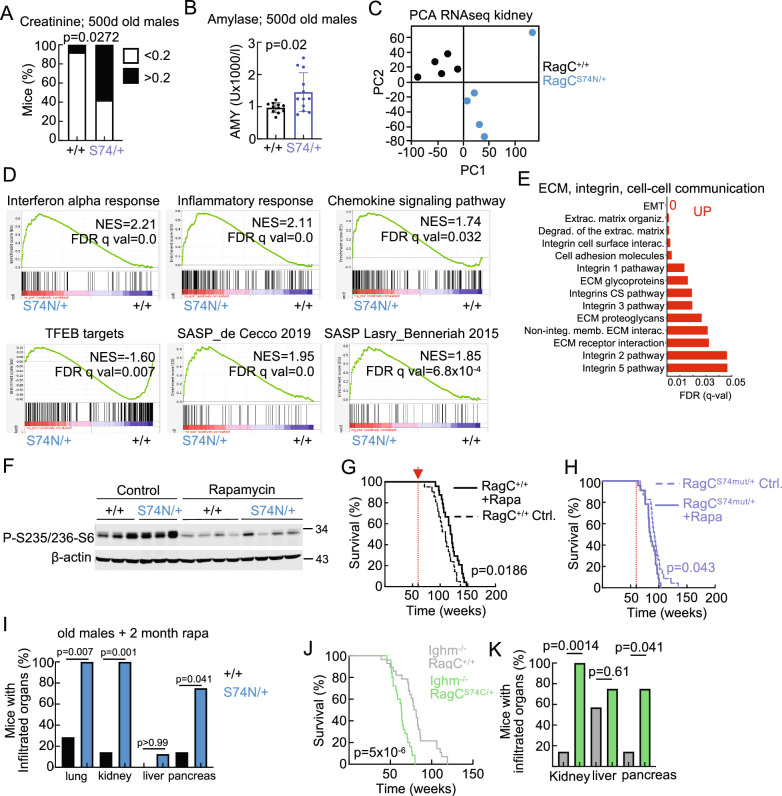
Old Rragc-mutant mice show an inflammaging phenotype. **a**. Percentage of mice with increased creatinine levels (>0.2 mg/dL) measured in blood from 18-month-old *Rragc*^+/+^ (n = 12) and *Rragc*^S74N + S74C/+^ (n = 12) male mice. Statistical significance was calculated by two-sided Fisher’s exact test. **b**. Amylase levels measured in blood from 18-month-old *Rragc*^+/+^ (n = 11) and *Rragc*^S74N + S74C/+^ (n = 12) male mice. Statistical significance was calculated by two-tailed Student’s t-test. Data are presented as mean values ± SD. **c**. Principal component analysis (PCA) from the RNA-seq performed from kidney samples from 18-month-old *Rragc*^+/+^ (n = 5) and *Rragc*^S74N/+^ (n = 5) mice. **d**. Gene set enrichment analysis (GSEA) related to inflammatory signatures, TFEB target genes, and genes involved in senescence-associated secretory phenotype in kidneys from 18-month-old *Rragc*^S74N/+^ (n = 5) versus *Rragc*^+/+^ (n = 5) mice. **e**. Graphical representation of the false discover rates (FDRs) from the indicated KEGG, Hallmark, REACTOME and Wikipathways gene sets enriched in kidneys from *Rragc*^S74N/+^ (n = 5) versus *Rragc*^+/+^ (n = 5) 18-month-old mice. **f**. Western blot of harvested kidneys from 16-month-old *Rragc*^+/+^ and *Rragc*^S74N/+^ male mice fed with control chow or with rapamycin-supplemented chow for 2 months. **g**. Kaplan–Meier survival curves of control (n = 21) and rapamycin-treated *Rragc*^+/+^ (n = 24) mice. Statistical significance was calculated with the log-rank test. **h**. Kaplan–Meier survival curves of control (n = 23) and rapamycin-treated *Rragc*^S74mut/+^ (n = 23) mice. Statistical significance was calculated with the log-rank test. **i**. Incidence of infiltrated inflammatory cells in the indicated tissues from 16-month-old *Rragc*^+/+^ (n = 7) and *Rragc*^S74N/+^(n = 8) mice treated with rapamycin for 2 months. Statistical significance was calculated by two-sided Fisher’s exact test. **j**. Kaplan–Meier survival curves of Ighm^-/-^; *Rragc*^+/+^ (n = 28) and Ighm^-/-^; *Rragc*^S74C/+^ (n = 25) mice. Statistical significance was calculated with the log-rank test. **k**. Incidence of infiltrated inflammatory cells in the indicated tissues from Ighm^-/-^; *Rragc*^+/+^ (n = 7) and Ighm^-/-^; *Rragc*^S74C/+^ (n = 8) mice analyzed at humane end point. Statistical significance was calculated by two-sided Fisher’s exact test. Source data

Quantification of peripheral blood mononuclear cells (PBMCs) from *Rragc*^+/+^ and *Rragc*^mut/+^ mice revealed another typical feature of old individuals, increased mature myeloid cells and decreased mature lymphoid populations^38,39^ (Fig. 2a,b). Histological examination of tissues from *Rragc*^S74N/+^ mice showed scattered but widespread foci of inflammation in kidney, pancreas, liver and lung (Fig. 2c–e). Moreover, inflammatory cytokines were significantly elevated in the peripheral blood from old *Rragc*^S74N/+^ mice (Fig. 2f). *Rragc*^S74N/+^ mice also presented elevated levels of creatinine and amylase in blood, markers of kidney and pancreas damage, respectively (Extended Data Fig. 2a,b). To identify transcriptomic changes reflecting underlying differences between old *Rragc*^+/+^ and old *Rragc*^S74N/+^ mice, we conducted bulk RNA sequencing (RNA-seq) from kidneys from ~16-month-old *Rragc*^+/+^ and *Rragc*^74N/+^ mice. Principal-component analysis (PCA) clustered samples by genotype (Extended Data Fig. 2c). Moreover, 1,368 genes were differentially expressed in old *Rragc*^S74N/+^ versus *Rragc*^+/+^ kidneys (792 upregulated and 576 downregulated in *Rragc*^S74N/+^; Supplementary Table 1). Among the most upregulated signatures in *Rragc*^S74N/+^ samples were inflammatory signatures such as interferon (IFN)γ and IFNα response, tumor necrosis factor (TNF) signaling and interleukin (IL)-6 signaling (Fig. 2g and Extended Data Fig. 2d). We also found chemokine-related signaling pathways (Fig. 2g and Extended Data Fig. 2d), integrins and extracellular matrix (ECM) remodeling signatures upregulated (Extended Data Fig. 2e). Moreover, SASP signatures^40,41^ were also enriched in *Rragc*^S74N/+^ samples (Extended Data Fig. 2d), in accordance with the increased SA-β-gal staining observed in the kidney (Fig. 1j,k). These results strongly suggest the existence of ‘inflammaging’, a basal and systemic myeloid inflammation associated with aging, and collectively support the occurrence of a phenotype resemblant of early onset of aging in mice with a mild increase in nutrient signaling.

RagC participates in a heterodimeric complex with RagA that binds mTORC1 in response to nutrients^42,43^, so we reasoned that pharmacological inhibition of mTORC1 with rapamycin could correct the phenotypic defects and the shortened longevity of *Rragc*^mut/+^ mice. In addition, rapamycin administration to ~1.5-year-old C57BL/6 mice significantly extends longevity^14^. Thus, we administered encapsulated rapamycin in food to 14-month-old mice, and at the same formulation as used elsewhere^14^. As expected, rapamycin-fed mice showed decreased phosphorylation of S6 in serines 235 and 236, and a canonical readout of suppressed mTORC1 activity in several organs, regardless of *Rragc* genotype (Extended Data Fig. 2f). Moreover, rapamycin administration led to a significant extension in longevity in *Rragc*^+/+^ mice (Extended Data Fig. 2g), but failed to extend survival in *Rragc*^mut/+^ (Extended Data Fig. 2h) mice and to decrease inflammation in organs from *Rragc*^mut/+^ (Extended Data Fig. 2i) mice. Collectively, these data suggest that chronic damage occurring before pharmacological inhibition of mTORC1 impaired a life-extending effect of rapamycin in *Rragc*^mut/+^ mice. In addition, the selective lack of effect of rapamycin in *Rragc*^mut/+^ mice points to rapamycin-resistant functions of an active RagC–mTORC1 axis, such as inhibition of TFEB family members^44,45^, playing a role in the shortened longevity of *Rragc*^mut/+^ mice.

Despite the lack of spontaneous lymphomas in *Rragc*^mut/+^ mice (Fig. 1f) and the lymphoid-to-myeloid inflammatory switch (Fig. 2a,b), we conceived that aberrant functions of B cells, or other immune cells, could be driving systemic health decline and death. To test whether aberrant B cell functions were an early driver of the inflammation and early death of *Rragc*^mut/+^ mice, we interbred *Rragc*^mut/+^ with *Ighm*^μMT/μMT^ mice, genetically deficient for mature B cells^46^, to generate *Rragc*^mut/+^ mice devoid of mature B cells. Overall survival of *Ighm*^μMT/μMT^ mice, regardless of the *Rragc* genotype, was shorter (Extended Data Fig. 2j) most likely due to their partial immunosuppression. Nevertheless, increased inflammation (Extended Data Fig. 2k) and premature death (Extended Data Fig. 2j) were still present in *Rragc*^mut/+^ mice in a B cell-deficient context. Together with the decreased B cell abundance in circulating blood from *Rragc*^mut/+^ compared to *Rragc*^+/+^ mice (Fig. 2b), these results show that increased inflammation and premature death are not a consequence of detrimental effects of aberrant B cell functions in *Rragc*^mut/+^ mice.

### Similarities between changes in *Rragc*^mut/+^ mice and physiological aging in mice and humans

**Fig. 3:**
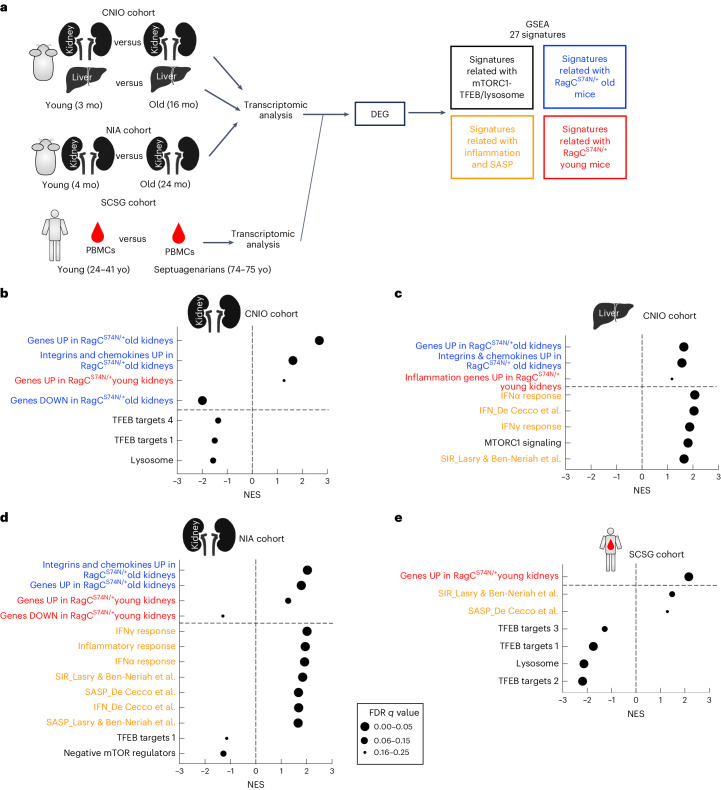
Similarities between changes in *Rragc*^mut/+^ mice and physiological aging in mice and humans. **a**, Experimental setup for the preranked GSEA performed in old and young wt mice from CNIO (kidney and liver) and NIA (kidney) cohorts, and the SCSG cohort (PBMCs from septuagenarians and young individuals) using 27 curated signatures related to mTORC1–TFEB and lysosomes (in black), signatures related to *Rragc*^S74N/+^ old mice (blue), signatures related to inflammation and SASP (orange) and signatures related to *Rragc*^S74N/+^ young mice (red). See Supplementary Table 2 for a detailed description of the signatures. **b**, Normalized enrichment score (NES) of indicated gene sets when comparing gene expression in old wt versus young wt kidneys from CNIO cohort. **c**, NES of indicated signatures when comparing gene expression in old wt versus young wt livers from CNIO cohort. **d**, NES of indicated signatures when comparing gene expression in old wt versus young wt kidney from NIA cohort. **e**, NES of indicated signatures when comparing gene expression in septuagenarians versus young PBMCs from the SCSG cohort. For **b**–**e**, a positive NES reflects enrichment of the gene set at the top of the ranked list and gene sets with a negative NES are overrepresented at the bottom of the gene list. The size of the bubble represents the FDR *q* value. Pictures of the liver and kidney were created with BioRender. Source data

The relevance of the Rag GTPase–mTORC1 pathway on aging is supported by genetic and pharmacological studies and is considered an important regulator of longevity across eukaryotes^2,3,16,17,47,48^. Nevertheless, because of the nature of the RagC mutations used herein, identified in patients with cancer and not in progeria patients, we sought to interrogate the relevance of our observations in physiological mouse and human aging. We analyzed the transcriptome of organs from young and old C57BL/6 mice from two independent cohorts: a cohort housed at the Spanish National Cancer Research Centre (CNIO) and an independent cohort house at the National Institute on Aging (NIA). To analyze these two independent datasets, we compiled a tailored list of gene signatures (Supplementary Table 2), including selected canonical gene sets from the Hallmark and KEGG databases, gene lists curated from previous literature on aging, senescence and inflammation^40,41^ and signatures of *TFEB* targets^49,50^. In addition, to specifically interrogate whether cellular processes downstream of activation of RagC are affected in normal aging, we curated lists of differentially expressed genes (DEGs) obtained from transcriptomic analyses (Extended Data Fig. 2c and Supplementary Table 1) of old and young organs from *Rragc*^S74N/+^ and wt (Fig. 3a) mice.

**Extended Data Fig. 3:**
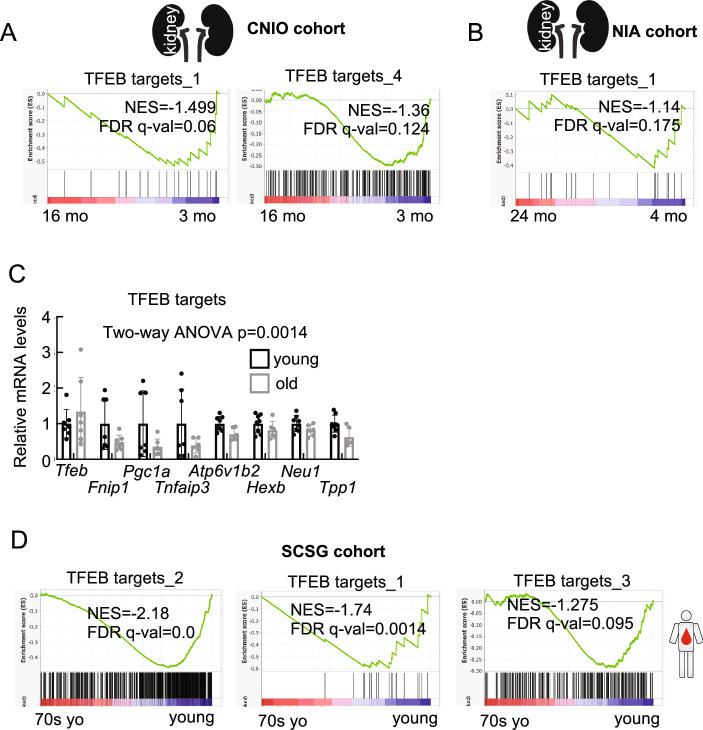
Similarities between changes in *Rragc*-mutant mice and physiological aging in mice and humans. **a**. Gene set enrichment analysis (GSEA) related to TFEB target genes in kidneys for CNIO cohort from 16-month-old wt (n = 5) versus 3-month-old (n = 4) mice. **b**. Gene set enrichment analysis (GSEA) related to TFEB target genes in kidneys for NIA cohort from 24-month-old wt (n = 5) versus 2-month-old (n = 5) mice. **c**. qRT–PCR analysis of TFEB target in kidney from 3-month-old wt (n = 8) and 16-month-old wt (n = 7) male mice. Statistical significance was calculated by two-way ANOVA. Data are presented as mean values ± SD. **d**. Gene set enrichment analysis (GSEA) related to TFEB target genes in PBMCs for the Spanish Centenarian Study Group (SCSG) cohort (Borras et al. 2016) from Septuagenarians (74-75 yr old) (n = 10) versus Young (24-41 yr old) (n = 10) individuals. Source data

In datasets from both CNIO and NIA, old wt mice show a striking enrichment of upregulated DEGs from *Rragc*^S74N/+^ young and old mice, whereas genes repressed in old and young *Rragc*^S74N/+^ mice are downregulated in old wt mice from CNIO and NIA cohorts. (Fig. 3b–d). These associations strongly support that both the early and late changes caused by activation of Rag GTPase signaling in mouse tissues strikingly overlap with the transcriptional changes that occur over time in naturally aged mice. Samples from naturally aged mice also exhibit upregulation of canonical signatures and previously published genes related to senescence, its secretory phenotype, inflammation, interferon (Fig. 3b–d), consistently with similar changes observed in *Rragc*^mut/+^ mice (Figs. 1l and 2g and Extended Data Fig. 2d,e) and indicating that processes occurring during physiological aging are exacerbated in, and not exclusive of, RagC-mutant mice. This similarity also applies if the comparison is conducted with genes related to inflammation and to integrins, senescence and chemokines. The direct consequence of activation of Rag GTPase signaling, namely activation of mTORC1 and suppression of TFEB activity and lysosomal biogenesis, which were seen in the transcriptomic analysis of *Rragc*^S74N/+^ mice (Extended Data Fig. 2d), are observed in samples from naturally aged CNIO and NIA cohorts (Fig. 3b–d and Extended Data Fig. 3a,b). Furthermore, we validated the expression by quantitative PCR with reverse transcription (qRT–PCR) of several TFEB targets and found a significant reduction in the transcript abundance of several TFEB targets in old wt kidneys compared to young wt kidneys (Extended Data Fig. 3c). Collectively, these analyses support that the Rag GTPase–mTORC1–TFEB–lysosome axis is deregulated during aging and suggest that this axis is of relevance for mammalian aging.

In addition to the analysis of mouse cohorts, we obtained the transcriptomic datasets from PBMCs from young adult and septuagenarian healthy individuals from the Spanish Centenarian Study Group (SCSG) cohort^51^. We applied the same curated signature analysis used above in search for overlaps between DEGs from RagC-mutant samples, canonical markers of aging and specific functions downstream of the Rag GTPases (TFEB and lysosome) in the septuagenarian cohort of this human study. The septuagenarian group exhibits a positive enrichment in the inflammatory signatures obtained from previous literature^40,41^ (Fig. 3e and Extended Data Fig. 3d) and notably, the upregulated genes from *Rragc*^S74N/+^ kidneys from young mice were enriched in septuagenarians compared to younger individuals. Moreover, samples from septuagenarians exhibit a significant depletion of the lysosome signature from KEGG and a significant depletion of signatures of TFEB targets. These positive and negative associations strongly support that changes in the Rag GTPase–mTORC1–lysosome occur in normal aging of both mice and humans.

### *Rragc*-mutant ‘reverse’ BM chimeras exhibit shortened longevity

**Fig. 4:**
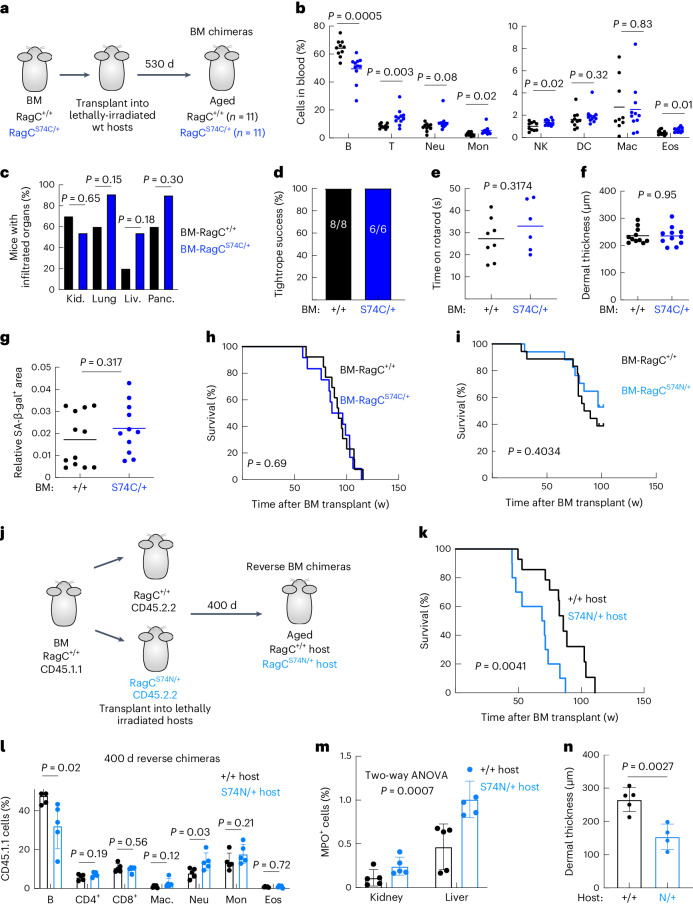
*Rragc*-mutant ‘reverse’ BM chimeras exhibit shortened longevity. **a**, Experimental setup for the generation of *Rragc*^+/+^ and *Rragc*^S74C/+^ BM chimeras. Aging features and inflammation were measured 18 months after transplantation. **b**, Percentage of cell populations in the blood of 18-mo-old BM-*Rragc*^+/+^ (*n* = 9) and BM-*Rragc*^S74C/+^ (*n* = 10) male mice. B, B cell; T, T cell; Mon, monocyte; NK, natural killer; Mac, macrophage; Eos, eosinophil. **c**, Incidence of inflammatory cells in tissues from 18-mo-old BM-*Rragc*^+/+^ (*n* = 10) and BM-*Rragc*^S74C/+^ (*n* = 11) mice. **d**, Tightrope assay in 18-mo-old BM-*Rragc*^+/+^ (*n* = 8) and BM-*Rragc*^S74C/+^ (*n* = 6) male mice. Bars represent the percentage of mice that passed the test. **e**, Time on rotarod test measured in the same mice as in **c**. **f**, Dermal thickness of back skin of 18-mo-old BM-*Rragc*^+/+^ (*n* = 11) and BM-*Rragc*^S74C/+^ (*n* = 11) male mice. **g**, Quantification of SA-β-gal^+^ area within the kidney area of 18-mo-old BM-*Rragc*^+/+^ (*n* = 12) and BM-*Rragc*^S74C/+^ (*n* = 11) mice. **h**, Kaplan–Meier survival curves of BM-*Rragc*^+/+^ (*n* = 13) and BM-*Rragc*^S74C/+^ (*n* = 12) mice. **i**, Kaplan–Meier survival curves of BM-*Rragc*^+/+^ (*n* = 9) and BM-*Rragc*^S74N/+^ (*n* = 20) mice. **j**, Experimental setup for the generation of reverse chimeric *Rragc*^+/+^ and *Rragc*^S74C/+^ mice. Readout 14 months after transplant of wt BM into *Rragc*^+/+^ and *Rragc*^S74N/+^ hosts. **k**, Kaplan–Meier survival curves of reverse BM-*Rragc*^+/+^ (*n* = 14) and BM-*Rragc*^S74N/+^ (*n* = 10) mice. **l**, Percentage of cell populations in the blood of 14-mo-old reverse BM-*Rragc*^+/+^ (*n* = 5) and BM-*Rragc*^S74N/+^ (*n* = 5) male mice. **m**, Quantification of IHC for myeloperoxidase in kidneys (left) and livers (right) collected from 14-mo-old reverse BM-*Rragc*^+/+^ (*n* = 5) and reverse BM-*Rragc*^S74N/+^ (*n* = 5) male mice. **n**, Dermal thickness measured in back skin of 14-mo-old reverse BM-*Rragc*^+/+^ (*n* = 5) and *Rragc*^*S74N*/+^ (*n* = 4) males. Statistical significance was assessed by two-tailed Student’s *t*-test (**b**,**e**–**g**,**l**,**n**); two-sided Fisher’s exact test (**c**,**d**); log-rank test (**h**–**k**); and two-way ANOVA (**m**). Data are presented as mean ± s.d. (**l**–**n**). Source data

**Extended Data Fig. 4:**
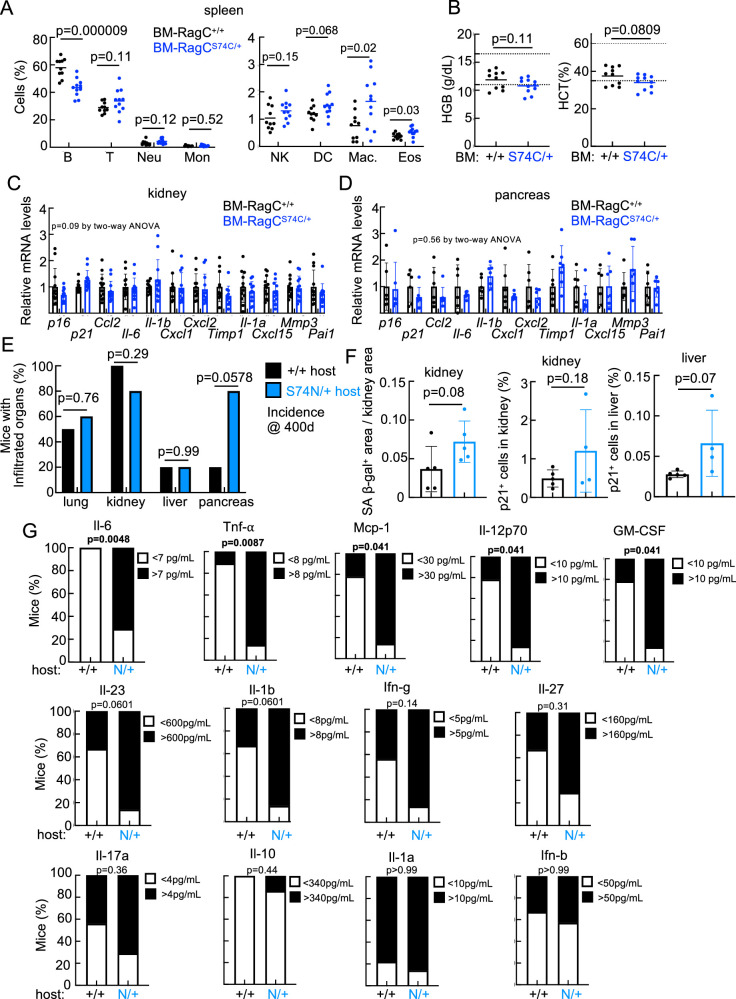
*Rragc-*mutant ‘reverse’ BM chimeras exhibit shortened longevity. **a**. Percentage of the indicated cell populations in the spleen of 18-month-old BM-*Rragc*^+/+^ (n = 9) and BM-*Rragc*^S74C/+^ (n = 10) male mice. (B: B cells, T: T cells, Neu: neutrophils, Mon: monocytes, NK: natural killers, DC: dendritic cells, Mac: macrophages and Eos: eosinophils. **b**. Hematological parameters (hemoglobin and hematocrit) measured in 18-month-old BM-*Rragc*^+/+^ (n = 10) and BM-*Rragc*^S74C/+^ (n = 11) male mice. **c**. qRT–PCR analysis of genes of the senescence-associated secretory phenotype (SASP) genes in kidneys from 18-month-old BM-*Rragc*^+/+^ (n = 12) and BM-*Rragc*^*S*74C/+^ (n = 12) male mice. Data are presented as mean values ± SD. **d**. qRT–PCR analysis of genes of the senescence-associated secretory phenotype (SASP) in pancreas from 18-month-old BM-*Rragc*^+/+^ (n = 12) and BM-*Rragc*^S74C/+^ (n = 12) male mice. Data are presented as mean values ± SD. **e**. Incidence of infiltrated inflammatory cells in the indicated tissues from 400 days-old *Rragc*^+/+^ (n = 5) and *Rragc*^S74N/+^(n = 5) male hosts. **f**. Quantification of SA-β-gal+ area within the kidney area (left), p21 in kidney (center) and liver (right) from 400 days-old *Rragc*^+/+^ (n = 5) and *Rragc*^S74N/+^(n = 5) male hosts. Data are presented as mean values ± SD. **g**. Quantification of inflammatory cytokines in sera from 390 days-old *Rragc*^+/+^ (n = 9) and *Rragc*^S74N/+^(n = 7) male hosts measured by Legendplex assay using flow cytometry. Statistical significance in a, b and f was calculated by two-tailed Student’s t-test. Statistical significance in c and d was calculated by two-way ANOVA. Statistical significance in e and g was calculated by two-sided Fisher exact test. Source data

B cells have a negligible effect in the premature death of *Rragc*^mut^ mice (Extended Data Fig. 2j). Nonetheless, the increased inflammation observed in old *Rragc*^S74N/+^ mice pointed to a bone marrow (BM)-derived population triggering the accelerated health decline in *Rragc*^mut/+^, so we sublethally irradiated wt C57BL/6 hosts and reconstituted their BM with cells obtained from BM of either *Rragc*^+/+^ or *Rragc*^mut/+^ mice (Fig. 4a), and monitored inflammation, markers of aging and overall survival. At 500 days, *Rragc*^mut/+^ BM chimeras had the same decrease in B cell lineage and partial increase in myeloid populations (Fig. 4b and Extended Data Fig. 4a) observed in full-body *Rragc*^mut/+^ mice (Fig. 2b), indicating that the lymphoid-to-myeloid switch observed in full-body *Rragc*^mut/+^ is BM-cell intrinsic. In contrast, BM chimeras failed to phenocopy the increased parenchymal inflammation (Fig. 4c), the decrease in hemoglobin and hematocrit (Extended Data Fig. 4b), the loss of neuromuscular coordination (Fig. 4d,e) and the dermal thinning (Fig. 4f) observed in full-body *Rragc*^mut/+^ mice. In addition, *Rragc*^mut/+^ BM chimeras did not show evidence of increased senescence (Fig. 4g and Extended Data Fig. 4c,d). More notably, and in contrast to the shortened lifespan of full-body *Rragc*^mut/+^ mice, the median and maximal lifespans of BM-*Rragc*^+/+^, BM-*Rragc*^S74C/+^ and BM-*Rragc*^S74N/+^ chimeras were identical (Fig. 4h,i). These transplantation experiments demonstrate that BM cells with increased nutrient signaling do not suffice to recapitulate the aging-like phenotype of mice with increased nutrient signaling.

We next decided to test whether the shortened lifespan of *Rragc*^mut/+^ mice was instead driven by parenchymal damage and so we conducted ‘reverse’ BM chimera reconstitution experiments, in which wt BM cells were used to reconstitute the hematopoietic system of young *Rragc*^+/+^ and *Rragc*^S74N/+^ sublethally irradiated hosts (Fig. 4j). Notably, *Rragc*^S74N/+^ hosts with wt BM had shortened longevity (Fig. 4k), lymphoid-to-myeloid switch (Fig. 4l), increased myeloid inflammation (MPO^+^ cells, Fig. 4m and Extended Data Fig. 4e), increased SA-β-gal with increase in systemic inflammatory cytokines (Extended Data Fig. 4f,g) and dermal thinning (Fig. 4n). Collectively, the BM transplantation experiments demonstrate that increased nutrient signaling in parenchymal cells, but not BM-derived cells, is sufficient to trigger the pro-aging-like phenotype of *Rragc*^mut^ mice.

### Old *Rragc*^S74N/+^ organs attract neutrophils

**Fig. 5:**
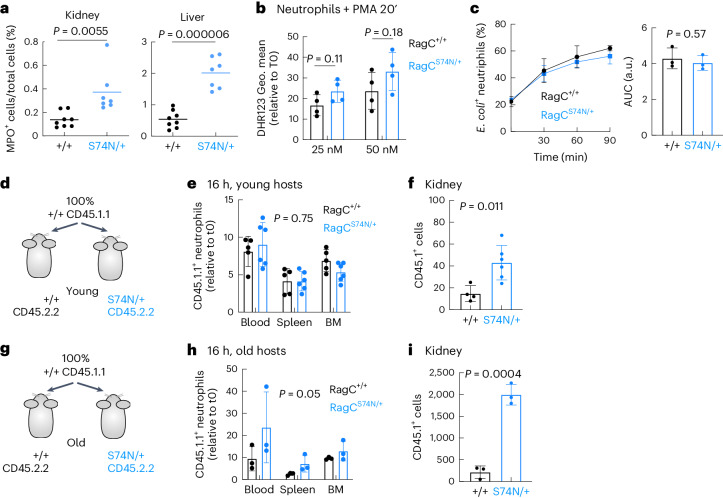
Old *Rragc*^S74N/+^ organs promote neutrophil infiltration. **a**, Quantification of IHC staining for myeloperoxidase in kidneys (left) and livers (right) collected from 18-mo-old *Rragc*^+/+^ (*n* = 8) and *Rragc*^S74N/+^ (*n* = 7) male mice. **b**, Reactive oxygen species production by neutrophils isolated from *Rragc*^+/+^ (*n* = 4) and *Rragc*^S74N/+^ (*n* = 4) BM as assessed by flow cytometry with DHR 123 after a 20-min treatment with PMA at the indicated concentrations. **c**, Kinetics of phagocytosis of GFP^+^ *E.* *coli* expressed as a percentage of GFP^+^ cells by neutrophils isolated from *Rragc*^+/+^ (*n* = 3) and *Rragc*^S74N/+^ (*n* = 3) BM. Data are presented as mean ± s.d. **d**, Experimental setup of the neutrophil adoptive transplant experiment. BM-derived neutrophils were isolated from 2–3-mo-old *Rragc*^+/+^ mice (CD45.1.1) and transplanted to 3-mo-old *Rragc*^+/+^ (CD45.2.2) and to 3-mo-old *Rragc*^S74N/+^ (CD45.2.2) male hosts. **e**, Quantification of wt CD45.1.1 neutrophils in the indicated tissues from CD45.2.2 *Rragc*^+/+^ (*n* = 5) and *Rragc*^S74N/+^ (*n* = 6) male hosts 16 h after the adoptive transplant. **f**, Quantification of CD45.1.1 wt neutrophils by IF in kidneys from CD45.2.2 *Rragc*^+/+^ (*n* = 4) and *Rragc*^S74N/+^ (*n* = 6) host males. **g**, Experimental setup of the neutrophil adoptive transplant experiment. BM-derived neutrophils were isolated from 3-mo-old Rragc^+/+^ mice (CD45.1.1) and transplanted to 12–14-mo-old *Rragc*^+/+^ (CD45.2.2) and *Rragc*^S74N/+^ (CD45.2.2) male hosts. **h**, Quantification of transferred wt CD45.1.1 neutrophils in the indicated tissues from CD45.2.2 *Rragc*^+/+^ (*n* = 3) and *Rragc*^S74N/+^ (*n* = 3) male hosts 16 h after adoptive transplant. **i**, Quantification of transferred wt CD45.1.1 neutrophils by IF in kidneys from CD45.2.2 *Rragc*^+/+^ (*n* = 3) and *Rragc*^S74N/+^ (*n* = 3) host males. Statistical significance was assessed by two-tailed Student’s *t*-test (**a**–**c**,**f**,**i**) and two-way ANOVA (**e**,**h**). Data in **b**,**c**,**e**,**f**,**h**,**i**, are presented as mean ± s.d. Source data

**Extended Data Fig. 5:**
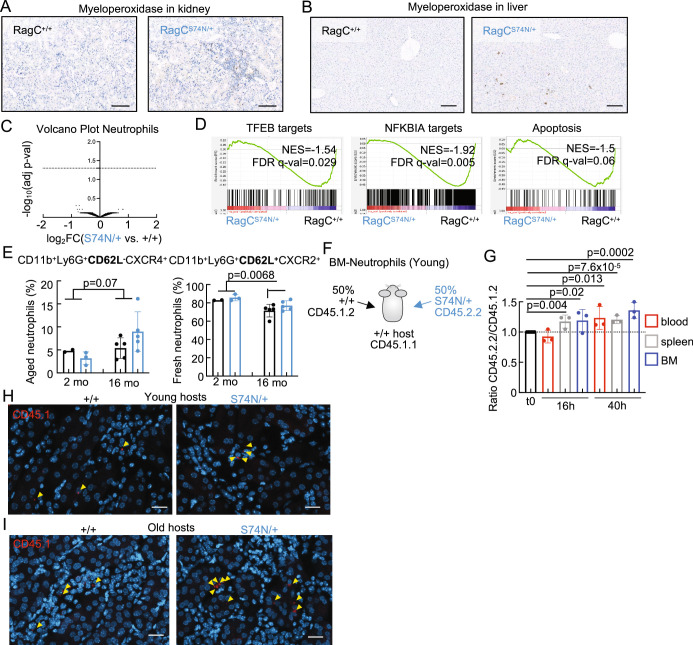
Old *Rragc*^S74N/+^ organs promote neutrophil infiltration. **a**. Representative IHC for myeloperoxidase (MPO) from kidneys harvested from 18-month-old *Rragc*^+/+^ and *Rragc*^S74N/+^ male mice. Bars represents 100 μm. **b**. Representative IHC for myeloperoxidase (MPO) from livers harvested from 18-month-old *Rragc*^+/+^ and *Rragc*^S74N/+^ male mice. Bars represents 100 μm. **c**. Volcano plot from RNA-seq performed in 6-8-week-old *Rragc*^S74N/+^ (n = 4) versus 6-8-week-old *Rragc*^+/+^ (n = 5) neutrophils isolated from BM. Differential expression was performed with DESeq, using a 0.05 FDR. **d**. Gene set enrichment analysis (GSEA) related to TFEB target genes, NFKBIA target genes and genes involved in apoptosis in 6-8-week-old *Rragc*^S74N/+^ (n = 4) versus 6-8-week-old *Rragc*^+/+^ (n = 5) neutrophils isolated from BM. **e**. Quantification of aged neutrophils (CD11b^+^/Ly6G^+^/CD62L^-^/CXCR4^+^, left) and fresh neutrophils (CD11b^+^/Ly6G^+^/CD62L^+^/CXCR2^+^, right) by flow cytometry in 2-month-old *Rragc*^+/+^ (n = 2) and *Rragc*^S74N/+^ (n = 3) male mice and in 16-month-old *Rragc*^+/+^ (n = 5) and *Rragc*^S74N/+^ (n = 5) male mice. Statistical significance was calculated by two-way ANOVA. Data are presented as mean values ± SD. **f**. Experimental setup of the neutrophil adoptive transplant experiment. BM-derived neutrophils were isolated from 10-week-old *Rragc*^+/+^ mice (CD45.1.2) and 10-week-old *Rragc*^S74N/+^ (CD45.2.2) and transplanted at 1:1 ratio to 16-week-old *Rragc*^+/+^ (CD45.1.1) hosts. **g**. Ratio of CD45.2.2/CD45.1.1 transplanted neutrophils in blood, spleen and BM 16 h (n = 3 biological replicates) and 40 h (n = 3 biological replicates) after adoptive transfer as described in f. Data are presented as mean values ± SD. Statistical significance was calculated by two-tailed Student’s t-test. **h**. Representative images of IF depicting CD45.1 positive cells in kidneys from young male hosts. Bars represent 20 μm. **i**. Representative images of IF depicting CD45.1 positive cells in kidneys from old male hosts. Bars represent 20 μm. Source data

While BM-derived cells from *Rragc*^mut/+^ mice *per se* do not shorten longevity, chronic inflammation and inflammatory damage may be a necessary player in response to organ damage caused by increased nutrient signaling in *Rragc*^mut/+^ mice and a critical contributor to their shortened lifespan. Of note, two of the most upregulated inflammatory cytokines in kidneys from old *Rragc*^mut/+^ mice were *Cxcl1* and *Cxcl2* (Fig. 1l), chemokines that promote activation and extravasation of neutrophils^52^. Thus, to quantitatively ascertain neutrophil infiltration, we quantified myeloperoxidase (MPO)-positive cells, a marker of neutrophil inflammation, by IHC and found a significant increase in neutrophil abundance in kidney and liver from old *Rragc*^mut/+^ mice (Fig. 5a and Extended Data Fig. 5a,b). Myeloid inflammation can be detrimental for organ homeostasis^53,54^, so to test whether *Rragc*^S74N/+^ neutrophils were cell-intrinsically dysfunctional and potential drivers of the health decline of *Rragc*^S74N/+^ mice, we purified BM-derived neutrophils from *Rragc*^+/+^ and *Rragc*^S74N/+^ mice and tested their cell-intrinsic ability to respond to activating stimuli in vitro. Treatment of purified neutrophils with the activating agent phorbol 12-myristate 13-acetate (PMA) resulted in similar activation and production of reactive oxygen species as indicated by the levels of the dihydrorhodamine (DHR) 123 probe (Fig. 5b) and a similar extent of activation-induced phagocytosis of *Escherichia* *coli* (Fig. 5c). Transcriptomic profiling of neutrophils purified from BM of five *Rragc*^+/+^ and five *Rragc*^S74N/+^ young mice, yielded minimal transcriptomic changes (Extended Data Fig. 5c), with zero DEGs and with only few significant enrichment of signatures, which included TFEB targets, NFKBIA targets and apoptosis in *Rragc*^+/+^ neutrophils (Extended Data Fig. 5d and Supplementary Table 1).

Neutrophils have a rapid turnaround time of approximately 1 day, and fresh and aged neutrophils can be distinguished by differential expression of cell-surface markers^55^. We wondered whether neutrophils were retained for longer periods of time and, thus, aged neutrophils would be increased in *Rragc*^S74N/+^ mice; however, aged neutrophils (CD11b^+^ Ly6G^+^, CD62L^−^ and CXCR4^+^) were equally increased in *Rragc*^+/+^ and *Rragc*^mut/+^ old mice and accordingly, fresh neutrophils were reduced in old mice of both genotypes (Extended Data Fig. 5e). Taken together, the transcriptomic, activation and cell-surface analyses in neutrophils are consistent with negligible intrinsic molecular and functional differences in neutrophils from *Rragc*^+/+^ and *Rragc*^S74N/+^ mice.

We next reasoned that parenchymal damage and chemoattractant signals from organs from *Rragc*^S74N/+^ mice could trigger neutrophil inflammation. To conclusively distinguish between neutrophil-intrinsic and non-intrinsic neutrophil responses in *Rragc*^S74N/+^ mice, we conducted a series of in vivo experiments with adoptive transfer of *Rragc*^+/+^ and *Rragc*^S74N/+^ neutrophils isolated from BM (experimental cartoon in Extended Data Fig. 5f). Competitive adoptive transfer of *Rragc*^+/+^; CD45.1.2 and *Rragc*^S74N/+^; CD45.2.2 in wt CD45.1.1 hosts resulted in a minimal, but significant increase in *Rragc*^S74N/+^; CD45.2.2 compared to transferred *Rragc*^+/+^; CD45.1.2 neutrophils (Extended Data Fig. 5g). We next interrogated whether wt neutrophils become differentially activated in *Rragc*^+/+^ versus *Rragc*^S74N/+^ hosts, so we transferred wt neutrophils (CD45.1.1) in young and old *Rragc*^+/+^ and *Rragc*^S74N/+^ CD45.2.2 hosts to assess differential behavior in differential environments (hosts of different *Rragc* genotype). When young wt neutrophils were transferred to young *Rragc*^+/+^ and *Rragc*^mut/+^ hosts (Fig. 5d), the relative abundance in the blood, spleen and BM 16 h post-injection was similar (Fig. 5e), but the extravasation of wt neutrophils to kidneys of young *Rragc*^S74N/+^ mice was significantly increased compared to extravasation of wt neutrophils in wt hosts (Fig. 5f and Extended Data Fig. 5h). Notably, the differential behavior of wt neutrophils was massively increased when transferred to old *Rragc*^+/+^ and *Rragc*^S74N/+^ recipients (Fig. 5g). The wt neutrophils in *Rragc*^S74N/+^ hosts were more abundant both in circulation and in the BM, spleen and kidney of old *Rragc*^S74N/+^ hosts (Fig. 5h,i and Extended Data Fig. 5i). Such differential persistence and extravasation of wt neutrophils in old *Rragc*^S74N/+^ hosts is consistent with damage signals stemming from old *Rragc*^mut/+^ organs that anomalously attract and activate myeloid cells. In turn, aberrantly behaving myeloid cells, through increased and sustained immune infiltration of *Rragc*^mut/+^ mice, may further contribute to perpetuate and worsen damage, senescence, SASP and further reinforce the chemoattractant signals for myeloid cells.

### Pro-inflammatory signals precede inflammation in young *Rragc*^S74N/+^ organs

**Fig. 6:**
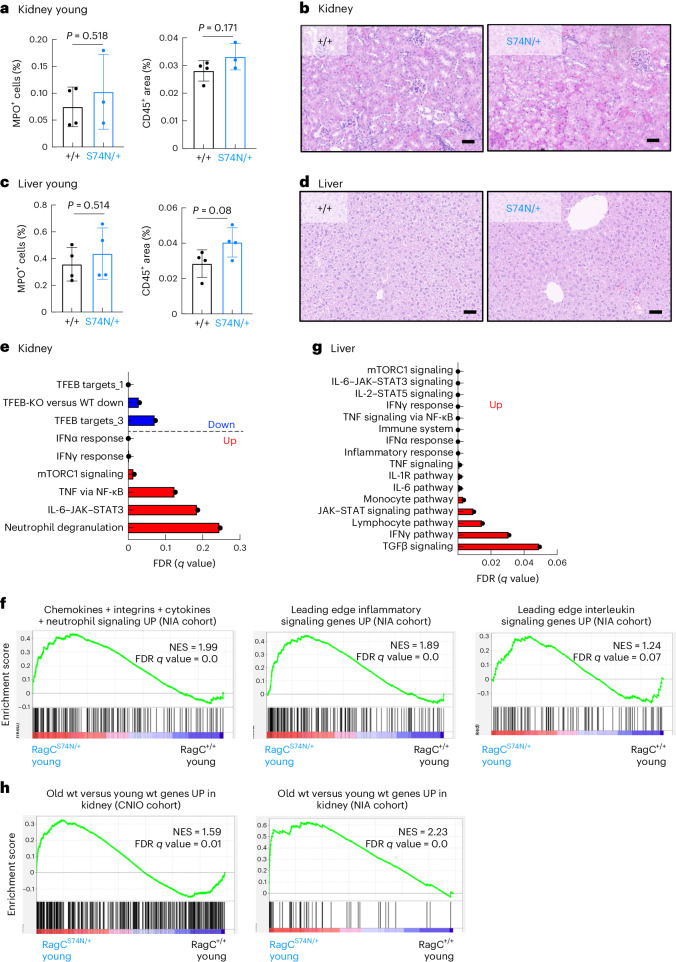
Pro-inflammatory signals precede inflammation in young *Rragc*^S74N/+^ organs. **a**, Quantification of IHC staining for myeloperoxidase (left) and CD45 (right) in kidneys collected from 2–4-mo-old Rragc^+/+^ (*n* = 4) and Rragc^S74N/+^ (*n* = 4) male mice. **b**, Representative H&E pictures from the same mice as in a, showing lack of inflammatory foci in kidney. Scale bars, 50 μm. **c**, Quantification of IHC staining for myeloperoxidase (left) and CD45 (right) in livers collected from 2–4-mo-old *Rragc*^+/+^ (*n* = 4) and *Rragc*^S74N/+^ (*n* = 4) male mice. **d**, Representative H&E pictures in the same mice as in **a**, showing lack of inflammatory foci in liver. Scale bars, 50 μm. **e**, Graphical representation of the FDRs from the indicated Hallmark, REACTOME, WikiPathways and curated gene sets enriched (red) and downregulated (blue) in kidneys from 3-mo-old *Rragc*^S74N/+^ (*n* = 5) versus *Rragc*^+/+^ (*n* = 5) mice. **f**, GSEA related to chemokines, integrins, cytokines, inflammatory pathways and IL signaling genes upregulated in old kidneys of NIA cohort in kidneys from 3-mo-old *Rragc*^S74N/+^ (*n* = 5) versus *Rragc*^+/+^ (*n* = 5) mice. See Supplementary Table 2 for further details of the gene sets. **g**, Graphical representation of the FDRs from the indicated KEGG, Hallmark, REACTOME and Biocarta gene sets enriched in livers from 2–3.5-mo-old *Rragc*^S74N/+^ (*n* = 4) versus *Rragc*^+/+^ (*n* = 4) mice. **h**, GSEA related to upregulated genes in old kidneys from CNIO (left) NIA cohort (right) in kidneys from 3-mo-old *Rragc*^S74N/+^ (*n* = 5) versus *Rragc*^+/+^ (*n* = 5) mice. See Supplementary Table 2 for further details of the gene sets. Statistical significance was assessed by two-tailed Student’s *t*-test (**a**,**c**). Data (**a**,**c**) are presented as mean ± s.d. Source data

**Extended Data Fig. 6:**
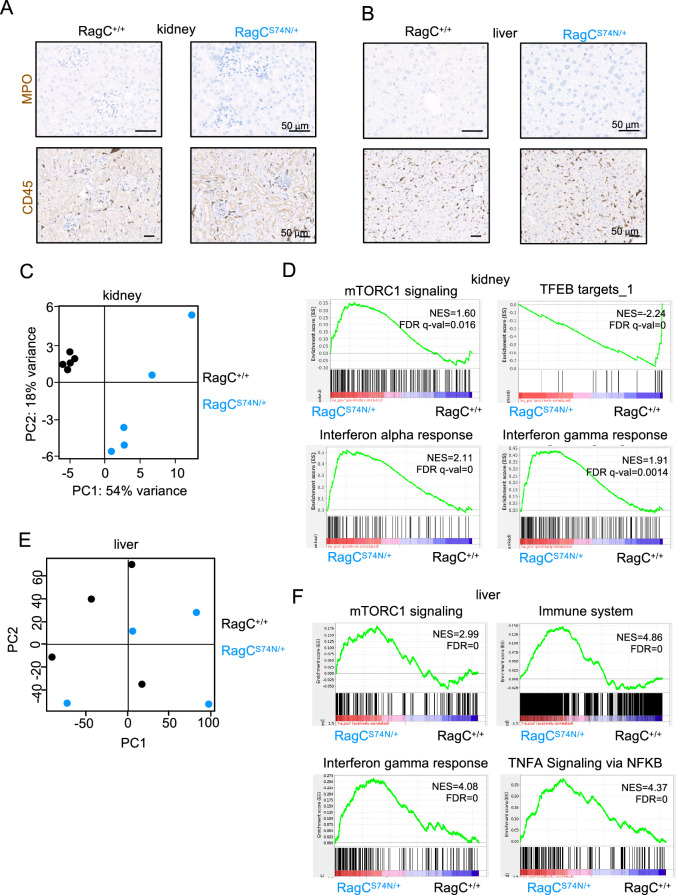
Pro-inflammatory signals precede inflammation in young *Rragc*^S74N/+^ organs. **a**. Representative images from anti-MPO and anti-CD45 immunostainings of kidneys harvested from 8-15-week-old *Rragc*^+/+^ and *Rragc*^S74N/+^ male mice. Bars represent 50 μm **b**. Representative images from anti-MPO and anti-CD45 immunostainings of livers harvested from 8-15-week-old *Rragc*^+/+^and *Rragc*^S74N/+^ male mice. Bars represent 50 μm. **c**. Principal component analysis (PCA) from the RNA-seq performed from kidney samples from 3-month-old *Rragc*^+/+^ (n = 5) and *Rragc*^S74N/+^ (n = 5) mice. **d**. Gene set enrichment analysis (GSEA) related to inflammatory signatures, TFEB target genes, and mTORC1 signaling associated genes in kidneys from 3-month-old *Rragc*^S74N/+^ (n = 5) versus *Rragc*^+/+^ (n = 5) mice. **e**. Principal component analysis (PCA) from the RNA-seq performed from liver samples from 3-month-old *Rragc*^+/+^ (n = 4) and *Rragc*^S74N/+^ (n = 4) mice. **f**. Gene set enrichment analysis (GSEA) related to inflammatory signatures and mTORC1 signaling associated genes in livers from 2–3.5-month-old *Rragc*^S74N/+^ (n = 4) versus *Rragc*^+/+^ (n = 4) mice.

To support the notion that myeloid cells inflict additional damage to organs in *Rragc*^mut/+^ mice in response to aberrant signals from peripheral organs from *Rragc*^mut/+^ mice, we conducted bulk RNA-seq from kidneys and livers from 2–4-month-old *Rragc*^+/+^ and *Rragc*^S74N/+^ mice, age in which myeloid inflammation and organ damage is not yet evident (Fig. 6a–d and Extended Data Fig. 6a,b). PCA clusters samples by genotype (Extended Data Fig. 6c), indicating that molecular differences are already evident at an early age and may underlie the extravasation of wt neutrophils observed in Fig. 5f,i. Moreover, in contrast to the absence of DEGs in purified neutrophils from *Rragc*^+/+^ versus *Rragc*^S74N/+^ young mice, 689 genes from the kidney were differentially expressed in young *Rragc*^*S*74N/+^ kidneys (335 upregulated and 354 downregulated; Supplementary Table 1). Among the top depleted signatures were Tfeb targets^28,50^ and mTORC1 activity and inflammation were among the top-enriched ones (Fig. 6e and Extended Data Fig. 6d). The changes in inflammatory genes and in chemokines and integrins identified in old *Rragc*^S74N/+^ kidneys from the NIA cohort (Supplementary Table 2) are already present in young *Rragc*^S74N/+^ organs, suggesting that these are early events during the aging process (Fig. 6f). Inflammatory signatures enriched in bulk RNA-seq were also evident in young livers (Fig. 6g, Extended Data Fig. 6e,f and Supplementary Table 1). Finally, the DEG genes between old and young wt mice from the CNIO and NIA cohorts (Supplementary Table 2) from Fig. 3 were found to be significantly enriched in young *Rragc*^S74N/+^ versus young *Rragc*^+/+^ mice (Fig. 6h), supporting again the molecular overlap of the early changes seen in young *Rragc*^S74N/+^ with those occurring in physiological aging.

### Myeloid cell depletion extends survival in *Rragc*^S74N/+^ mice

**Fig. 7:**
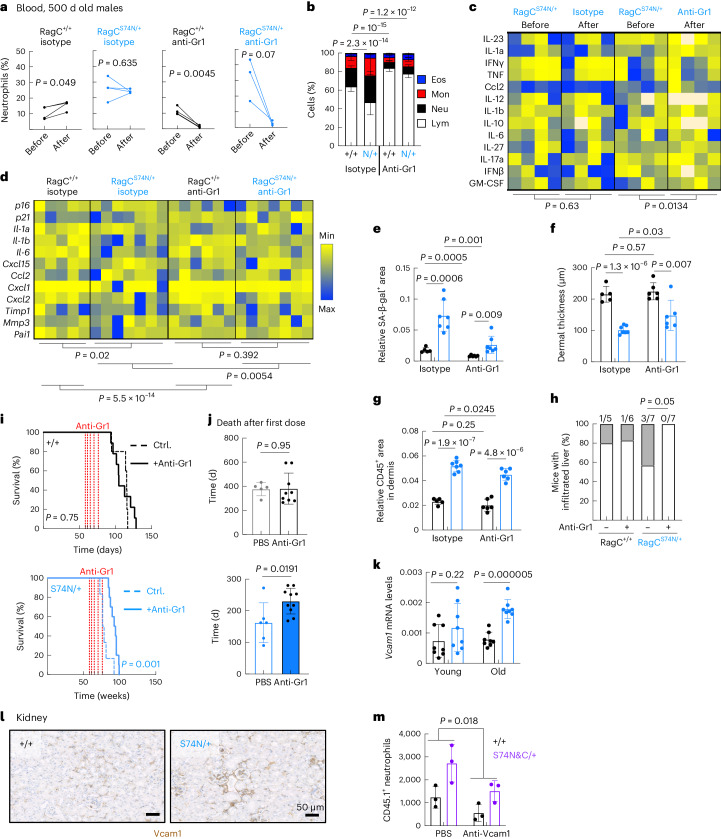
Myeloid cell depletion corrects markers of inflammaging and extends survival in *Rragc*^S74N/+^ mice. **a**, Quantification of neutrophils in peripheral blood before and after a 5-day treatment with anti-Gr1 or isotype control antibody from isotype-treated *Rragc*^+/+^ (*n* = 4), anti-Gr1-treated *Rragc*^+/+^ (*n* = 4), isotype-treated *Rragc*^S74N/+^ (*n* = 4) and anti-Gr1-treated *Rragc*^S74N/+^ (*n* = 3) 24-mo-old male mice. **b**, WBC count in isotype-treated *Rragc*^+/+^ (*n* = 5) and *Rragc*^S74N/+^ (*n* = 7), anti-Gr1-treated *Rragc*^+/+^ (*n* = 6) and *Rragc*^S74N/+^ (*n* = 7) 24-mo-old male mice. **c**, Quantification of cytokines in sera from isotype-treated *Rragc*^S74N/+^ (*n* = 4) and anti-Gr1-treated *Rragc*^S74N/+^ (*n* = 4) 24-mo-old male mice in a 5-day treatment measured by Legendplex assay. **d**, qRT–PCR analysis of SASP genes in the kidneys from isotype-treated *Rragc*^+/+^ (*n* = 5) and *Rragc*^S74N/+^ (*n* = 7), anti-Gr1-treated *Rragc*^+/+^ (*n* = 6) and *Rragc*^S74N/+^ (*n* = 7) 18-mo-old male mice. **e**, Quantification of SA-β-gal^+^ area within the kidney area of isotype-treated *Rragc*^+/+^ (*n* = 5) and *Rragc*^S74N/+^ (*n* = 7), anti-Gr1-treated *Rragc*^+/+^ (*n* = 6) and *Rragc*^S74N/+^ (*n* = 7) of 18-mo-old mice. **f**, Dermal thickness in back skin of the same mice as in **e**. **g**, Quantification of IHC of CD45^+^ cells in skin from the same mice as in **f**. **h**, Incidence of infiltrated inflammatory cells in livers from mice in **e**. **i**, Kaplan–Meier survival curves of control (*n* = 5) and anti-Gr1-treated (*n* = 9) *Rragc*^+/+^ mice (left) and controls (*n* = 6) and anti-Gr1-treated (*n* = 10) *Rragc*^S74N/+^ mice (right). **j**, Survival since the first dose of anti-Gr1 of the mice in **k**. **k**, qRT–PCR analysis of *Vcam1* in kidneys of young (4-mo-old) and old (18-mo-old) *Rragc*^+/+^ (young, *n* = 8; old, *n* = 8) and *Rragc*^S74N/+^ (young, *n* = 8; old, *n* = 8) male mice. **l**, Representative IHC staining of Vcam1 in kidney from 18-mo-old *Rragc*^+/+^ and *Rragc*^S74N/+^ mice. **m**, Quantification of transferred wt CD45.1.1 neutrophils by IF in kidneys from CD45.2.2 host males treated with PBS (*Rragc*^+/+^ (*n* = 3); *Rragc*^S74mut/+^ (*n* = 3)) or anti-Vcam1 (*Rragc*^+/+^ (*n* = 3); *Rragc*^S74mut/+^ (*n* = 3)). Statistical significance was assessed by one-tailed Student’s *t*-test (**a**); two-tailed Student’s *t*-test (**e**–**g**, **j**,**k**); two-sided Fisher’s exact test (**h**); two-way ANOVA (**b**–**d**,**m**); and log-rank test (**l**). Data (**b**,**e**–**g**,**j**,**k**,**m**) are mean ± s.d. Source data

**Extended Data Fig. 7:**
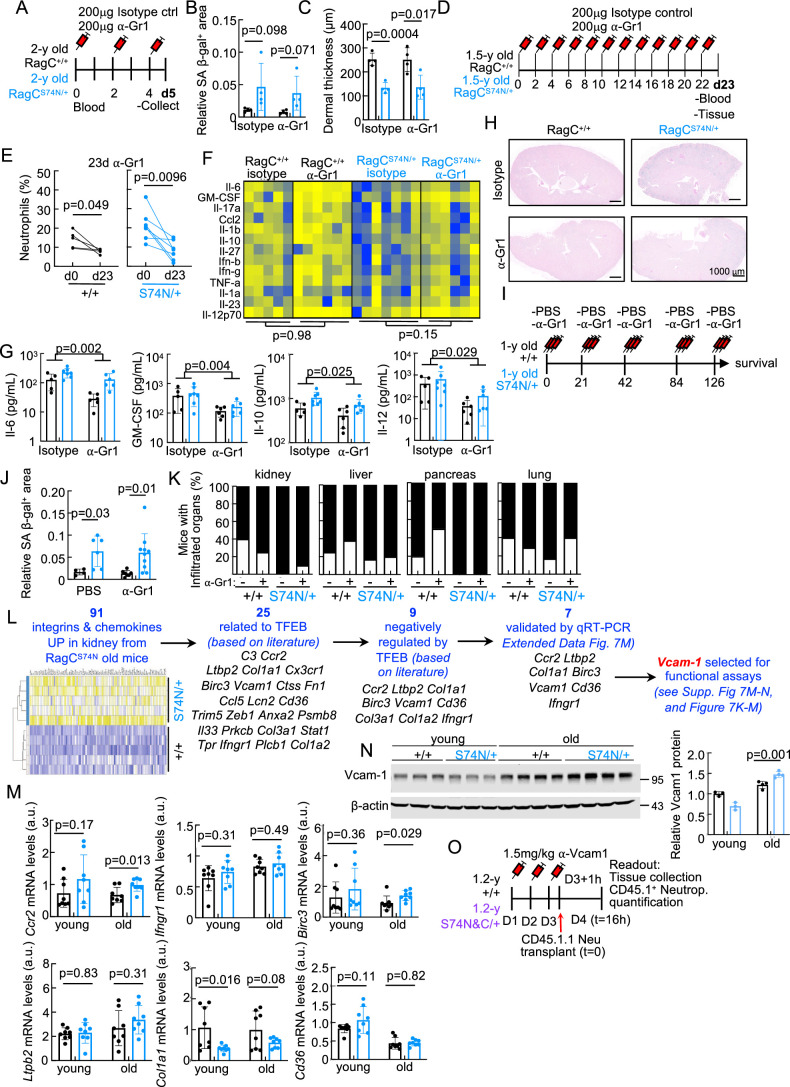
Myeloid cell depletion corrects markers of inflammaging and extends survival in *Rragc*^S74N/+^ mice. **a**. Experimental setup for α-Gr1 acute treatment. *Rragc*^+/+^ and *Rragc*^S74N/+^ male mice were treated with 200μg of α-Gr1 or isotype at day 0, 2 and 4. At 5d, mice were euthanized and blood and tissues were collected. **b**. Quantification of SA-β-gal^+^ area within the kidney area of 2 y old isotype-treated *Rragc*^+/+^ (n = 5), anti-Gr1 treated *Rragc*^+/+^ (n = 7), isotype-treated *Rragc*^S74N/+^ (n = 6), anti-Gr1-treated *Rragc*^S74N/+^ (n = 7) 2 y old male mice. Data are presented as mean values ± SD. **c**. Dermal thickness measured in back skin of isotype-treated *Rragc*^+/+^ (n = 5), anti-Gr1-treated *Rragc*^+/+^ (n = 7), isotype-treated *Rragc*^S74N/+^ (n = 6), anti-Gr1-treated *Rragc*^S74N/+^ (n = 7) 2-year-old male mice. Data are presented as mean values ± SD. **d**. Experimental setup for three-week anti-Gr1 treatment. One-and-a-half-year-old male mice were treated with 200μg of anti-Gr1 or isotype control every other day for 23d, and mice were sacrificed and blood and tissues were processed. **e**. Percentage of neutrophils measured in blood before and after a 23-day treatment of anti-Gr1 treated *Rragc*^+/+^ (n = 5) and anti-Gr1-treated *Rragc*^S74N/+^ (n = 7) 18-m-old male mice. Statistical significance was calculated by one-tailed Student’s t-test. **f**. Quantification of inflammatory cytokines in sera from isotype-treated *Rragc*^+/+^ (n = 5), anti-Gr1-treated *Rragc*^+/+^ (n = 6), isotype-treated *Rragc*^S74N/+^ (n = 7), anti-Gr1-treated *Rragc*^S74N/+^ (n = 6) 18-month-old male mice after a 23-day treatment. **g**. Concentration of the indicated cytokines in sera from isotype-treated *Rragc*^+/+^ (n = 5), anti-Gr1 treated *Rragc*^+/+^ (n = 6), isotype-treated *Rragc*^S74N/+^ (n = 7), anti-Gr1 treated *Rragc*^S74N/+^ (n = 6) 1.5-year-old male mice. Data are presented as mean values ± SD. **h**. Representative pictures of SA β-gal stains from isotype-and anti-Gr1-treated mice. **i**. Experimental setup for extended anti-Gr1 survival experiment. One-year-old *Rragc*^+/+^ and *Rragc*^S74N/+^ male mice were treated either with PBS or anti-Gr1 every other day for two weeks, followed by a resting period of 3 weeks. This regimen was repeated five times so mice received either PBS or anti-Gr1 antibody intermittently during approximately 6 months. **j**. Quantification of SA-β-gal^+^ area within the kidney area of mice under the regime described in j. PBS-treated *Rragc*^+/+^ (n = 4), anti-Gr1-treated *Rragc*^+/+^ (n = 7), PBS-treated *Rragc*^S74N/+^ (n = 6), anti-Gr1 treated *Rragc*^S74N/+^ (n = 10) males. Kidneys were harvested at humane end point. Data are presented as mean values ± SD. **k**. Incidence of infiltrated inflammatory cells in the indicated tissues from PBS-treated *Rragc*^+/+^ (n = 5), anti-Gr1-treated *Rragc*^+/+^ (n = 7), PBS-treated *Rragc*^S74N/+^ (n = 6) and anti-Gr1-treated *Rragc*^S74N/+^ (n = 10) mice. Statistical significance was calculated by two-sided Fisher’s exact test. **l**. Heatmap of integrins and chemokines upregulated in kidney from *Rragc*^+/+^ (n = 5) and *Rragc*^S74N/+^ (n = 5) mice. Out of 91 genes, 25 genes related to TFEB were selected based on literature and 9 were negatively regulated by TFEB (based on literature); 7 out of 9 were validated by qRT–PCR and finally, *Vcam1* was selected for functional assays. **m**. qRT–PCR analysis of integrins and chemokines negatively regulated by TFEB in kidneys of young (4-month-old) and old (18-month-old) *Rragc*^+/+^ (young, n = 8; old, n = 8) and *Rragc*^S74N/+^ (young, n = 8; old, n = 8) male mice. Data are presented as mean values ± SD. **n**. Immunoblot and quantification for Vcam1 from 4-month-old *Rragc*^+/+^ (n = 3 biological independent samples) and *Rragc*^*S74N*/+^ (n = 3 biological independent samples) and 18-month-old *Rragc*^+/+^ (n = 4 biological independent samples) and *Rragc*^*S74N*/+^ (n = 4 biological independent samples) male mice. Data are presented as mean values ± SD. **o**. Experimental setup for α-Vcam1 treatment and neutrophil adoptive transplant. Mice were treated with PBS or 1.5 mg/kg of α-Vcam1 three consecutive days and one hour after the last dose, CD45.1.1 BM-isolated neutrophils were transplanted to 16-24-month-old *Rragc*^+/+^ (CD45.2.2) and *Rragc*^S74N/+^ (CD45.2.2) male and female hosts. Statistical significance in a, b, j, m, and n was calculated by two-tailed Student’s t-test. Statistical significance in f and g was calculated by two-way ANOVA. Source data

Excessive myeloid inflammation in response to organ damage can be pathogenic and inflict additional damage paracrinally, so even if *Rragc*^S74N/+^ myeloid cells were not intrinsically abnormal, they could elicit further organ deterioration if activated by parenchymal signals. To determine whether secondary myeloid inflammation contributes to the health decline of *Rragc*^mut/+^ mice, we acutely depleted neutrophils and monocytes by intraperitoneal injection of a blocking antibody for Gr1 (ref. ^56^), in 24-month-old mice. Three anti-Gr1 injections within 1 week (Extended Data Fig. 7a) were sufficient to eliminate circulating neutrophils in both *Rragc*^+/+^ and *Rragc*^S74N/+^ mice (Fig. 7a), while also partially correcting the skewed PBMC populations from old *Rragc*^S74N/+^ mice (Fig. 7b). Moreover, acute treatment reduced inflammatory cytokines in blood from *Rragc*^S74N/+^ mice (Fig. 7c), without correcting aging features such as SA-β-gal activity (Extended Data Fig. 7b) and dermal thickness (Extended Data Fig. 7c). A 3-week depletion of Gr1^+^ cells resulted in sustained reduction of neutrophils (Extended Data Fig. 7d,e), partial correction of inflammatory markers (Extended Data Fig. 7f,g) and the downregulation of the expression of inflammatory genes in livers from *Rragc*^mut/+^ mice (Fig. 7d). Notably, at this 3-week time point, a significant amelioration of several of the markers of aging were detected in *Rragc*^mut/+^ mice: significant decrease in SA-β-gal activity in the kidney (Fig. 7e and Extended Data Fig. 7h, which is also evident in *Rragc*^+/+^ mice), expansion of the dermal thickness and decrease in dermal inflammation (Fig. 7f,g) and a reduction of liver infiltration (Fig. 7h) in anti-Gr1 treated, but not isotype-antibody treated, old *Rragc*^S74N/+^ mice.

We next sought to evaluate the long-term effect of controlling myeloid inflammation. This effort constituted an experimental challenge because repetitive injections of anti-Gr1 results in a feedback response that results in the maturation of neutrophils and other myeloid cells with reduced expression of the Gr1 receptor, becoming refractory to the intervention^57^. Thus, we designed an intermittent regime of five cycles of anti-Gr1 injections, followed by resting periods, over a total of 6 months, to enable transient but recurrent depletion of myeloid cells (Extended Data Fig. 7i). Starting at approximately the same age as that of the cohorts used for pharmacological inhibition of mTORC1 with rapamycin (Extended Data Fig. 2g,h), long-term injections of anti-Gr1 antibody significantly extended the median and maximal lifespan of *Rragc*^S74N/+^ mice (Fig. 7i,j; control, 73.36 weeks; anti-Gr1, 93.14 weeks), a ~23% extension of overall survival and a remarkable ~40% extension if measured since the day of the first dose of anti-Gr1 (Fig. 7i,j). Histopathological examination and quantification of SA-β-gal activity in the kidney revealed no differences between control mice and mice treated with anti-Gr1 antibodies at a humane end point, suggesting that the suppression of myeloid inflammation delays, rather than changes, the ultimate causes of death of *Rragc*^mut/+^ (Extended Data Fig. 7j,k). Altogether, the results of different regimes of myeloid cell depletion show that controlling inflammation under pathologic nutrient signaling, even at old ages, is an efficacious intervention to extend longevity.

To identify potential myelo-attractant genes differentially expressed in organs of *Rragc*^*S*74N/+^ mice, and to assess their functional effect on the abnormal infiltration, we first selected all integrins and chemokines significantly increased in the bulk RNA-seq analysis from kidney samples (Extended Data Fig. 7l). From those 91 DEGs, we conducted a literature search to select those reported to be controlled by TFEB, and from those 25, we selected 9 that were reported to be negatively regulated by TFEB, as its transcriptional activity is suppressed by increased RagC–mTORC1 signaling. We validated seven of them by qPCR (Extended Data Fig. 7m) and selected Vcam1 for the functional validation of its involvement in myeloid infiltration in old *Rragc*^*S*74N/+^ mice based on reported function^58,59^ and preliminary analysis of *Rragc*^*S*74N/+^ samples by western blot and IHC (Fig. 7k,l and Extended Data Fig. 7n). Thus, we repeated the wt neutrophil transfer experiments (as in Fig. 5g) with the addition of α-Vcam1-blocking antibodies and quantified neutrophil extravasation (Extended Data Fig. 7o). Supporting our hypothesis, blockade of Vcam1 inhibited wt neutrophil extravasation in old *Rragc*^+/+^ and *Rragc*^S74N/+^ mice (Fig. 7m).

Collectively, our work provides strong support for a two-step pro-aging effect of increased nutrient signaling, involving parenchymal damage and a secondary myeloid inflammation that can be therapeutically mitigated even at old age.

## Discussion

The connections of the mTORC1 signaling pathway and aging across eukaryotes are extensive. Pharmacological inhibition of mTOR results in extended longevity in yeast, worms, flies and mice^2^. Moreover, genetic approaches to study mTORC1 signaling in aging have been undertaken in yeast, worms and flies, but equivalent genetic approaches in mice have been limited by the occurrence of specific detrimental phenotypes that precluded the analysis of time-dependent health decline. *Pten*-deficient and *Tsc*1/2-deficient mice die in utero and heterozygous deletion of *Pten* or *Tsc*1 or *Tsc*2 result in the development of tumors following loss of heterozygosity^18,20–23^. While these findings were invaluable for understanding developmental biology, metabolic physiology and cancer biology, systemic and other tissue-specific models of upregulation of mTORC1 activity yielded fewer lessons for the understanding of the processes underlying aging driven by increased mTORC1 activity^60,61^. Partial loss-of-function approaches have been undertaken, including heterozygous mice for several components of mTORC1 (ref. ^17^) and a hypomorphic mTOR mutant^16^, which revealed that the links between mTORC1 activity and longevity exceed the systemic metabolic effects of this cascade.

Here we report a mouse model of a systemic increase in Rag GTPase–mTORC1 activity leading to a pro-aging-like phenotype. We propose that this phenotype is revealed in *Rragc*^mut/+^ mice precisely because of a mild increase in mTORC1 activity. In contrast to the tissue-specific deleterious pathologies seen in strains with stronger activation of the mTORC1 pathway, a moderate and systemic increase in nutrient signaling allows mice to survive long enough and with minimal phenotypic alterations to manifest time-dependent decline in organ function.

Notably, the transcriptomic changes occurring in *Rragc*^mut/+^ organs from young and old mice exhibit a large overlap with the transcriptomic changes that take place during physiological aging of wt mice, thus supporting the extrapolation of the conclusions obtained from the molecular and cellular analyses of *Rragc*^mut/+^ mice for ‘normal’ mammalian aging and positing that the nutrient–Rag GTPase–mTORC1 axis is involved in mammalian longevity. Moreover, based on the overlap of transcriptomic signatures from *Rragc*^mut/+^ mice and septuagenarians (Fig. 3), the involvement of this axis could be extrapolated also to human aging.

*Rragc*^mut/+^ mice show surprisingly decreased spontaneous tumor development. A simplistic yet counterintuitive interpretation of this finding would be that activating mutations in RagC have tumor suppressive functions, in spite of being bona fide oncogenic for B cells. In this regard, recent evidence points to a liability of activating mutations in the nutrient signaling cascade in conditions of limited nutrient availability in cultured cells^62^.

Neither B cells nor inflammation per se drive the shortened lifespan of mice with increased nutrient signaling. Instead, peripheral organ damage results in secondary myeloid inflammation. In turn, this inflammation perpetuates and worsens the peripheral organ damage, as reported in other physiological settings^63^. The pharmacological intervention to suppress neutrophil inflammation with an anti-Gr1 antibody (Fig. 7), together with the in vitro activation of neutrophils, strongly support the notion that myeloid cells are unlikely to be cell-autonomously activated in an aberrant manner by RagC mutations, but rather respond to signals such as Cxcl1 and Cxcl2 and Vcam1, emanating from prematurely damaged organs of *Rragc*^mut/+^ mice.

The identification of inflammation as a mediator of the shortened longevity by increased nutrient signaling evidences the irreplaceable value of mouse genetics for understanding the inter-organ communications and interactions that mediate the complexity of processes that precipitate the evolution of aging, which can be arguable not entirely captured by approaching this in other model organisms.

The largely beneficial effects of depletion of myeloid cells contrasts with the negligible effect of pharmacological inhibition of mTORC1 with rapamycin administered in the same formulation, dose, regime and starting at an age similar to that reported elsewhere^14^. The effect of rapamycin in extending lifespan is proportional to the duration of the dosing^14,15^. Hence, it is conceivable that rapamycin extended the survival of wt mice because their remaining lifespan when dosing started was long enough to allow rapamycin to exert its longevity-promoting effect, but was insufficient in the short-lived *Rragc*^mut/+^ mice. This is a valid interpretation of our data, but is at odds with the strong protective effect of the anti-Gr1 intervention when administered at the same age as of the rapamycin-treated *Rragc*^mut/+^ mice. An alternative interpretation is that organ damage by increased mTORC1 activity in *Rragc*^mut/+^ was already extensive, irreparable and not tunable by pharmacological inhibition of mTORC1 at an advanced age. Moreover, rapamycin had barely any effect in the chronic inflammation of old *Rragc*^mut/+^, so it supports the concept that inflammation secondarily occurring in response to organ damage is a relevant player in the process of nutrient signaling-driven aging. Unlike what occurs in lymphomas^29^, another potential explanation for negligible effect of rapamycin is related to the program executed by the RagC–mTORC1 axis during aging. Compelling new evidence^64,65^ states that RagC-dependent activation of mTORC1 results in preferential phosphorylation of members of the TFEB family. Indeed, our transcriptional profiles of organs from *Rragc*^mut/+^ support a strong suppression of Tfeb transcriptional activity and, notably, phosphorylation of TFEB family members by mTORC1 is particularly insensitive to the allosteric mode of action of rapamycin. Thus, if *Rragc*^mut/+^ cells have impaired Tfeb activity due to sustained phosphorylation by mTORC1, minimal effect of rapamycin would be expected. The mitigation of myeloid infiltration by the blockade of Vcam1 in our myeloid transplantation setting supports the relevance of the RagC–mTORC1–Tfeb axis, as Tfeb activity suppresses the expression of Vcam1 (ref. ^66^). Finally, activating mutations in RagC may also shorten longevity by yet-to-be-identified functions independent of canonical mTORC1 activity.

Our dissection of the determinants of the aging-like phenotype in mice with increased nutrient signaling reveals that chronic inflammation is actionable as a potential therapeutic control of the health decline. Many aging-related diseases have a positive correlation with BMI^67^, which also associates with mild chronic inflammation; conversely, dietary restriction (DR) is an extremely efficacious intervention to extend healthy aging and longevity in mammals. While DR and mTORC1 inhibition are molecularly distinguishable^5,68^, some degree of epistasis between DR and mTORC1 exists^3^, so presumably DR acts at least partially through the modulation of the Rag GTPase–mTORC1 signaling axis.

## Methods

### Mice

All animal procedures carried out at the CNIO were approved by the CNIO-ISCIII Ethics Committee for Research and Animal Welfare (CEIyBA) and by the Autonomous Community of Madrid (certificates PROEX 15/18, PROEX 215.17, 139.1/22 and PROEX 225.7/22). Mouse work at the NIA was approved by the Animal Care and Use Committee of the NIA in Baltimore (277-TGB-2022). *Ighm*^μMT^ mice^46^ were obtained from The Jackson Laboratories (stock 002288). Mice were housed under specific-pathogen-free conditions at 22 °C and 45–65% humidity and with 12-h dark–light cycles. Mice were fed with a standard chow diet (Harlan Teklad 2018). All mice were observed weekly by trained personnel. Upon signs of morbidity, mice were closely inspected daily until application of humane end point criteria (http://dels.nas.edu/global/ilar/Guide). For BM reconstitution experiments, host mice were sublethally irradiated with two 4.5-Gy doses within a 4-h period following by injection of BM cells. Rapamycin was supplied encapsulated in chow diet at 42 ppm (Rapamycin Holdings and Purina Lab Diet). For anti-Gr1 treatment, mice were injected intravenously with 200–400 μg of anti-Gr1 antibody (BioXcell, clone RB6-8C5, BE0075) or isotype control (BioXcell, clone LTF-2, BE0090). For anti-Vcam1 treatment, mice were injected intravenously with 1.5 mg kg^−1^ of anti-Vcam1 antibody (BioXcell, clone M/K-2.7, BE0027-25MG) or PBS.

### Generation of *Ragc*^S74N^ Crispr-edited KI mice

Blastocysts were injected with the Cas9 mRNA, a single-guide RNA targeting the *Rragc* locus and a single-stranded DNA oligonucleotide containing the desired mutation flanked by 40–60 bases homologous to the sequence adjoining the DNA double-strand break, as carried out previously^29,69^. Following clone selection, genotyping was performed by PCR and restriction fragment length polymorphisms or Sanger sequencing. We utilized a gRNA with the following sequence (in minus orientation): CGGCAAATCCTCCATCCAGA. The repair oligonucleotide contained the AAC mutations plus five additional silent mutations (CGGCAAAAACAGTATCCAAAAA).

### Metabolic parameters

Blood was collected from the submandibular vein. Hemoglobin and hematocrit were determined by a blood cell counter (CVM LaserCell) and blood urea nitrogen, creatinine and amylase were measured using VetScan rotors (Abaxis, 500-0038-25).

### Behavioral tests

For the tightrope assay, mice were placed on a bar of a circular section (60 cm long and 1.5 cm in diameter) and the test was considered successful when a mouse remained on the bar for 60 s in at least one of five consecutive trials^70^. The rotarod tests were performed in a Panlab LE 8200 using acceleration from 4–40 rpm in a period of 120 s. The time to fall was recorded and an average of three trials was used^71^. Mice were previously trained for three consecutive days.

### Measurement of blood pressure

Blood pressure was measured using a noninvasive tail-cuff method with the CODA system (CODA-HT2, Kent Scientific Corporation)^72^. Mice were habituated for three consecutive days.

### Detection of cytokines in serum

Sera from mice were collected and analyzed with a cytokine assay LEGENDplex Mouse Inflammation Panel (13-plex) (BioLegend, 740446). Data were collected by flow cytometry (BD FACSCanto II) and cytokines were quantified with a LEGENDplex Data Analysis Software Suite.

### SA-β-gal stain in tissues

The SA-β-gal stain was performed using a Senescence β-Galactosidase Staining kit (Cell Signaling Technology, 9860). Kidneys were fixed in 1× Fixative solution overnight at room temperature (RT) and then incubated for 30 h at 37 °C. Tissues were washed twice with 50% ethanol and twice with 70% ethanol. Kidneys were embedded in paraffin blocks and nuclear fast red stain was performed.

### Isolation of neutrophils for transplants

Neutrophils were purified from CD45.1.1 mouse BM using a 65% Percoll gradient (GE HealthCare, 17-0891-01) and red blood cells were lysed using Erythrocyte Lysis Buffer (QIAGEN, 79217). Then, 2–5 × 10^6^ neutrophils were transferred to recipient mice bled 2 min after transplant to determine the input number of CD45.1.1 neutrophils.

### Signaling in MEFs

Subconfluent cell cultures were rinsed and placed in RPMI without amino acids (US Biological, R8999-04A), supplemented with 10% dialyzed FBS and all 20 amino acids for 1 h. After that, cells were rinsed three times and then placed in RPMI without amino acids supplemented with 10% dialyzed FBS.

### Immunofluorescence in MEFs

MEFs were seeded on 12-mm-diameter coverslip in a 12-well plate (150,000 cells per well), one day before the experiment. After 1 h of the indicated treatment, cells were fixed with 8% paraformaldehyde in PBS (EMB, 15710) for 10 min at RT. Cells were permeabilized with 0.05% Triton X-100 (Sigma, T9284) in PBS for 5 min at RT, followed by three washes with PBT (PBS + Tween 0.1% (Sigma, P7949)). After blocking in 5% goat serum (Sigma, G9023) in PBT for 1 h, slides were incubated with TFEB antibody (1:100 dilution, Bethyl Laboratories*,* A303-673A) for 1 h at RT, washed 3× with PBT and incubated with goat anti-rabbit IgG (H+L) Cross-Adsorbed Secondary Antibody, Alexa Fluor 488 (1:300 dilution, Life Technologies, A11008) for 1 h at RT. Coverslips were then washed three times and incubated with 4,6-diamidino-2-phenylindole (DAPI; Sigma, D9542) for 5 min. After washing three times with PBT, coverslips were mounted with Fluoromount-G (Bionova Cientifica, 0100-01).

### Staining and flow cytometry analysis

Mononuclear cells were isolated from mouse blood, spleen or bone marrow (BM). Cells were separated by crushing the spleens through a 70-μm mesh (Corning) in ice-cold PBS + 0.1% BSA + 3 mM EDTA, and red blood cells were lysed using Erythrocyte Lysis Buffer. Cells from blood were isolated after red blood cells lysis and BM cells were isolated by flushing cells from the tibia and femur and collecting them in ice-cold PBS + 0.1% BSA + 3 mM EDTA. Cell staining was performed on ice in PBS + 0.1% BSA + 3 mM EDTA after a step of incubation with Fc-block Reagent (anti-CD16/CD32, BD Pharmigen, 553142). Macrophages were identified as F4/80^+^/SSC^hi^. NK cells were identified as F4/80^−^B220^−^/CD3^−^/NK.1.1^+^. Dendritic cells were identified as F4/80^−^B220^−^/CD3^−^/NK.1.1^−^/MHCII^+^/CD11c^+^. Neutrophils were identified as F4/80^−^/B220^−^/CD3^−^/NK.1.1^−^/MHCII^−^/CD11c^−^/CD11b^+^/Ly6G^+^. Monocytes were identified as F4/80^−^/B220^−^/CD3^−^/NK.1.1^-^/MHCII^−^/CD11c^−^/CD11b^+^/Ly6G^−^/Ly6C^l^°^w or high^. Eosinophils were identified as F/480^−^/B220^−^/CD3^−^/NK.1.1^-^/MHCII^−^/CD11c^−^/CD11b^+^/Ly6G^−^/Ly6C^med^. Aged neutrophils^55^ were identified as CD11b^+^/Ly6G^+^/CD62L^−^/CXCR4^+^. Fresh neutrophils were identified as CD11b^+^/Ly6G^+^/CD62L^+^/CXCR2^+^. Flow cytometry analyses included BD LSRFortessa or BD FACSCanto II cell analyzers, running BD FACSDiva software (BD Biosciences). FlowJo software (v.9.8.1 and v.10; TreeStar) was used for data analyses and plot rendering.

### Assessment of neutrophil function

#### Reactive oxygen species production

Neutrophils were purified from BM using a 65% Percoll gradient (GE HealthCare, 17-0891-01). Then, 5 × 10^5^ neutrophils were plated and incubated with 2.5 μg ml^−1^ DHR 123 (Molecular Probes, D23806) and 25 or 50 nM PMA (Sigma-Aldrich, P1585) for 20 min at 37 °C and 5% CO_2_. After incubation, cells were washed with ice-cold PBS + 0.1% BSA + 3 mM EDTA, stained for cell-surface markers and analyzed by flow cytometry^73^.

#### Phagocytosis of *E.coli*^GFP^

Neutrophils were purified from BM using a 65% Percoll gradient. Then, 5 × 10^5^ neutrophils were incubated with GFP-expressing *E.* *coli* in a ratio of 1:100 for 30–90 min at 37 °C. Cells were collected and stained for cell surface markers and analyzed by flow cytometry.

### Immunoblotting

Cells were rinsed once with ice-cold PBS and lysed in ice-cold lysis buffer (50 mM HEPES, pH 7.4, 40 mM NaCl, 2 mM EDTA, 1.5 mM sodium orthovanadate, 50 mM NaF, 10 mM pyrophosphate, 10 mM glycerophosphate, 1% Triton X-100 and one tablet of EDTA-free complete protease inhibitors (Roche) per 25 ml). Small pieces of frozen tissues were homogenized using a FastPrep-24 5G tissue homogenizer. Lysates were cleared by centrifugation at maximum speed for 10 min. Protein extracts were denatured by the addition of sample buffer, resolved by SDS–PAGE and analyzed by immunoblotting. Data collection was performed using Odyssey Infrared Imaging System (Application Software v.3.0.30. LI-COR Biosciences, NDP.view2 software). In addition, analyses were performed according to standard procedures using FIJI ImageJ v.1.53.

### Subcellular protein fractionation in tissues

Tissues were extracted with a Subcellular Protein Fractionation kit for tissues (Thermo Scientific, 87790), adjusting the volumes to the sample mass. After protein extraction, the cytosolic and nuclear fractions were quantified using BCA (Thermo Scientific, 23222). Then, 30 µg cytoplasmic and 30 µg nuclear fraction from the kidney and 25 µg cytoplasmic and 8 µg nuclear fraction from the heart were used for immunoblotting.

### RNA extraction, cDNA synthesis and qRT–PCR

Total RNA was extracted from tissues with TRItidy (Panreac Applichem, A4051,0200) and using an RNeasy kit from QIAGEN (74106). RNA was extracted from cells with a TRItidy and Picopure RNA Isolation kit (Arcturus, Applied Biosystems, 12204-01). RNA was treated with DNase (QIAGEN, 79254) before cDNA synthesis. To perform cDNA synthesis, 1 μg RNA was retrotranscribed using SuperScript IV VILO Master Mix (Invitrogen, 11756500). Quantitative real-time PCR was performed using GoTaq qPCR Master Mix (Promega, 6001) in a QuantStudio 6 Flex Real-Time thermocycler (Applied Biosystems). Data were analyzed by the change-in-threshold (2^−ΔCT^) method, using β-actin as the housekeeping reference gene. Primers were designed using Primer3 Software (http://bioinfo.ut.ee/primer3-0.4.0/).

### Gene expression profiling of neutrophils, liver and kidney from CNIO cohorts

Neutrophils (CD45^+^, CD11b^+^ and Ly6G^+^) were sorted from *Rragc*^S74N/+^ and *Rragc*^+/+^ BM cells in a BD FACSAria Ilu (Becton Dickinson) and InFlux (Cytopeia-Becton Dickinson) cell sorters. For the liver and kidney RNA-seq, total RNA samples (300–500 ng) were processed into cDNA sequencing libraries with the QuantSeq 3′ mRNA-Seq Library Prep kit (FWD) for Illumina (Lexogen, 015). For neutrophil preparations (RNA range 16–180 ng), a unique molecular identifier second-strand synthesis module (Lexogen, 081) was used. Library generation was initiated by reverse transcription with oligodT priming and a second-strand synthesis was performed from random primers. Primers from both steps contained Illumina-compatible sequences. Libraries were completed by PCR and sequenced on an Illumina NextSeq 550 System (with v.2.5 reagent kits).

The resulting reads were analyzed with the nextpresso^74^ pipeline: sequencing quality was checked with FastQC v.0.11.7 (https://www.bioinformatics.babraham.ac.uk/projects/fastqc/); reads were aligned to the mouse genome (GRCm39 for neutrophils and young and old kidney RNA-seq and GRCm38 in the case of young liver RNA-seq) with TopHat2 (ref. ^75^) using Bowtie^76^ and SAMtools^77^, allowing three mismatches and 20 multi hits. The GENCODE vM25 gene annotation for GRCm38 was used; read counts were obtained with HTSeq^78^ v.0.6.1 (–stranded = yes), using the mouse gene annotation from GENCODE (gencode.vM26.GRCm39.Ensembl103 for neutrophil and young and old kidney RNA-seq, gencode.vM20.GRCm38.Ensembl95 for young liver RNA-seq). Differential expression was performed with DESeq^79^, using a 0.05 false discovery rate (FDR). Gene Set Enrichment Analysis (GSEA) Preranked^80^ was used to perform gene set enrichment analysis for several gene signatures on a preranked gene list, setting 1,000 gene set permutations. Only those gene sets with significant enrichment levels (FDR *q* value < 0.25) were considered.

### Gene expression profiling of kidneys from NIA cohort

Four-month-old and 24–26-month-old C57LB/6J mice were killed in the morning under fed conditions and kidneys were snap frozen in liquid N_2_ and stored at −80 °C. For RNA isolation, 50 mg kidney tissue was weighed, resuspended in cold TRIzol Reagent (Invitrogen Life Technologies) and homogenized in a TissueLyser (QIAGEN). Downstream steps for RNA isolation were carried out automatically on a QIACube by using the RNAeasy Plus kit (QIAGEN).

For kidney RNA-seq, total RNA samples (750 ng) were processed into cDNA sequencing libraries with the SMARTer Stranded Total RNA Sample Prep kit - HI Mammalian kit and unique dual-indexed PCR primers for amplification were used (Takara Bio). Paired-end sequencing was performed for 103 cycles in an Illumina Novaseq 6000 sequencer and real-time analysis generated the base-call files, which were de-multiplexed and converted to standard FASTQ files using the bcl2fastq program (v.2.20.0.422). The quality of reads was first assessed using fastqc v.0.11.8 (https://www.bioinformatics.babraham.ac.uk/projects/fastqc/). Next, reads were trimmed using bbduk (part of the bbtools package; https://jgi.doe.gov/data-and-tools/software-tools/bbtools/). Reads were then aligned to the mm10 reference genome and ENSEMBL annotation v.89 using STAR v.2.7.8a in two-pass mode^81^. The BAM files generated by STAR were sorted and indexed using SAMtools v.1.9 (ref. ^77^). To generate a count matrix, featureCounts v.2.0.1 (ref. ^82^) was utilized. An overall quality control assessment was then performed using multiqc v.1.9 (ref. ^83^). To perform differential gene expression analysis, the genes in the count matrix were filtered by requiring at least ten samples to have a count greater than 10. Finally, DESeq2 v.1.40.2 (ref. ^79^) was used for analysis. The apeglm method^84^ was used for visualization and ranking. Genes with an adjusted *P* value <0.05 were considered significant.

### Histological and immunohistochemical analyses of mouse tissues

Tissue samples were fixed in 10% neutral buffered formalin, paraffin-embedded, cut at 3 μm, mounted in superfrost plus slides and dried overnight. Consecutive rehydrated sections were stained with hematoxylin and eosin (H&E) and IHC reactions were performed in an automated platform (Ventana Discovery XT). Antigen retrieval was first performed with the appropriate pH buffer (CC1m, Ventana, Roche) and endogenous peroxidase was blocked (peroxide hydrogen at 3%). Slides were then incubated with rat monoclonal anti-p21 (clone 291HUGO; 1:10 dilution; CNIO Monoclonal Antibodies Core Unit, also Abcam, 107099), rabbit polyclonal MPO (1:1,250 dilution, DAKO, A0398), rabbit polyclonal CD45 (clone D3F8Q, 1:200 dilution, Cell Signaling Technology, 70257), rabbit monoclonal Vcam1 (clone D2T4N, 1:200 dilution, Cell Signaling Technology, 32653) and TFEB (1:1,500 dilution, Bethyl, A303-673A). After addition of the primary antibody, slides were incubated with the visualization systems (Omni Map anti-rabbit, Ventana, Roche) conjugated with horseradish peroxidase. IHC reactions were developed using 3, 30-diaminobenzidine tetrahydrochloride (DAB) (Chromo Map DAB, Ventana, Roche; DAB Dako) counterstained with Carazzi’s hematoxylin. For p21 and MPO quantification, whole slides were acquired with a slide scanner (AxioScan Z1, Zeiss). Different images from different slides were chosen for quantification program training (Zen Blue software package, Zeiss) and an appropriate script for p21 and MPO quantification was created: positivity was evaluated in one phase (phase 1, positive cells) and compared to tissue area (phase 2, tissue area). After training and script optimization, the quantification program was run and results were exported as Excel files with scoring data for each TIFF file. For TFEB quantification, whole slides were acquired with a slide scanner (AxioScan Z1, Zeiss). For each mouse, whole kidney sections were selected and analyzed using Qupath software^85^. The deep-learning-based segmentation method StarDist^86^ was used to perform cell segmentation and to classify the cells as positive or negative for TFEB staining, followed by TFEB subcellular location assessment.

### CD45.1.1 immunofluorescence

Mouse kidneys were collected and fixed in periodate-lysine-paraformaldehyde overnight at 4 °C. Samples were washed twice and dehydrated successively in 10%, 20% and 30% sucrose for 2 h each at 4 °C with shaking. Tissues were frozen in OCT Compound Cryostat Embedding Medium (Tissue Tek, 4583) using dry ice and cut into 10-µm sections in Superfrost Plus Adhesion Microscope Slides (Epredia, J1810AMNZ) using a cryostat (Leica CM1950, Leica Biosystems). For immunofluorescence (IF) staining, sections were permeabilized in TRIS 0.1 M in PBS + 1% Triton X-100 for 1 h at RT and blocked with anti-mouse CD16/CD32 (1:100 dilution, Fc-Block, BD Pharmigen, 553142) for 1 h at RT. To identify transferred neutrophils, tissues were stained with AF647-labeled anti-CD45.1 antibody (1:100 dilution, BioLegend, 110720) in TRIS 0.1 M in PBS + 1% BSA + 0.3% Triton X-100 overnight at 4 °C. DAPI (5 μg ml^−1^; Sigma-Aldrich, D9542) was used for cell nuclei staining. Sections were mounted with ProLong(R) Gold Antifade Reagent (Cell Signaling Technology, 9071S) and analyzed with a confocal microscope (LSM 900, Zeiss Microscopy). Neutrophil quantification was performed manually using ZEN lite software v.3.6 (Zeiss Microscopy).

### Statistics and reproducibility

The *n*, indicating the total number of animals per group, as well as the definition of center, dispersion and precision measures are indicated in each figure and figure legend. Unless otherwise stated, a two-tailed Student’s *t*-test or chi-squared test was performed as described in the figures. Error bars represent s.d. Survival in mouse experiments was represented with Kaplan–Meier curves and significance was estimated with a log-rank test. Statistical analyses were performed with Prism v.9 software (GraphPad). For the heatmap of integrins and chemokines in old kidneys, expression patterns were displayed using the pheatmap package (RRID SCR_016418) in R software (https://cran.rproject.org/web/packages/pheatmap/index.html), with expression data from RNA-seq, normalized by *z*-score. Hierarchical clustering was performed using Euclidean correlation distance. No statistical methods were used to predetermine sample sizes. Data distribution was assumed to be normal but this was not formally tested. Mice were randomly assigned to different treatments and conditions. Mouse studies were performed in a blinded fashion; mice had numbered IDs but no information on genotypes was available in the cage cards. Genotypes were verified at the analysis step. The genotype of the mouse was not disclosed to the pathologist. During survival experiments, some mice needed to be killed due to animal discomfort not related to aging pathologies (dermatitis and a corneal ulcer) according to the guidelines for Humane Endpoints for Animals used in Biomedical Research and were excluded from the study. Experiments in Fig. 1a were performed three times. The experiment in Extended Data Fig. 1e was performed once using four independent replicates. The experiment in Extended Data Fig. 2f was performed once using 3–4 independent replicates per genotype and treatment. The experiment in Fig. 1b and Extended Data Fig. 1g was performed once in each tissue using three independent biological replicates per genotype. The experiments in Figs. 1c–n, 2c,f,g, 3b–e, 4c–n, 5a–c, 6a,c,e–h, 7a–k,m and Extended Data Figs. 1f, j–r, 2a–e,g–k, 3c, 4b–g, 5c,e,g, 6c–f, 7b–k,m were performed once. The experiment in Extended Data Fig. 1i was performed once using four independent replicates per genotype. The experiments in Fig. 1n and Extended Data Fig. 7n were performed once with 3–4 independent replicates for genotype and age. The experiments in Fig. 2a,b were performed four times. The experiment in Figs. 4b and 5e–i and Extended Data Fig. 4a were performed twice.

### Ethics approval on human samples

The ethics approval certificate for the analysis of data from previous work^51^ was issued on 5 February 2010 by the Ethics Committee of the Hospital Universitario de la Ribera de Alzira.

### Reporting summary

Further information on research design is available in the Nature Portfolio Reporting Summary linked to this article.

### Supplementary information

Supplementary Information Supplementary Information on gating strategy for FACS.

Reporting Summary

Supplementary Table 1 Lists of DEGs, GSEA, signatures, primers and antibodies.

### Source data

Source Data Fig. 1 Excel tabs with source data for Fig. 1.

Source Data Fig. 2 Excel tabs with source data for Fig. 2.

Source Data Fig. 3 Excel tabs with source data for Fig. 3.

Source Data Fig. 4 Excel tabs with source data for Fig. 4.

Source Data Fig. 5 Excel tabs with source data for Fig. 5.

Source Data Fig. 6 Excel tabs with source data for Fig. 6.

Source Data Fig. 7 Excel tabs with Source data for Fig. 7.

Source Data Extended Data Fig. 1 Excel tabs with source data for Extended Data Fig. 1.

Source Data Extended Data Fig. 2 Excel tabs with source data for Extended Data Fig. 2.

Source Data Extended Data Fig. 3 Excel tabs with source data for Extended Data Fig. 3.

Source Data Extended Data Fig. 4 Excel tabs with source data for Extended Data Fig. 4.

Source Data Extended Data Fig. 5 Excel tabs with source data for Extended Data Fig. 5.

Source Data Extended Data Fig. 7 Excel tabs with source data for Extended Data Fig. 7.

## Data Availability

Sequence data that support the findings of this study have been deposited in the Gene EXpression Omnibus under accession codes GSE255148, GSE255864, GSE221283, GSE221284, GSE221285 and GSE221286. The human transcriptomic data from the SCSG study are deposited in ArrayExpress under identifier E-MTAB-9988. Other data supporting the findings of this study are available in Source Data. All other data supporting the findings of this study are available as source data and from the corresponding author upon request.
