# Supplementary material for: A mild increase in nutrient signaling to mTORC1 in mice leads to parenchymal damage, myeloid inflammation and shortened lifespan
Source: Nat Aging. 2024 Jun 7;4(8):1102–20. doi: 10.1038/s43587-024-00635-x (PMC11333293; doi:10.1038/s43587-024-00635-x)

nature aging

Supplementary information https://doi.org/10.1038/s43587-024-00635-x

# A mild increase in nutrient signaling to mTORC1 in mice leads to parenchymal damage, myeloid inflammation and shortened lifespan

In the format provided by the authors and unedited

## Gating strategy Figure 2B, 4B, Supplementary Figure  4A

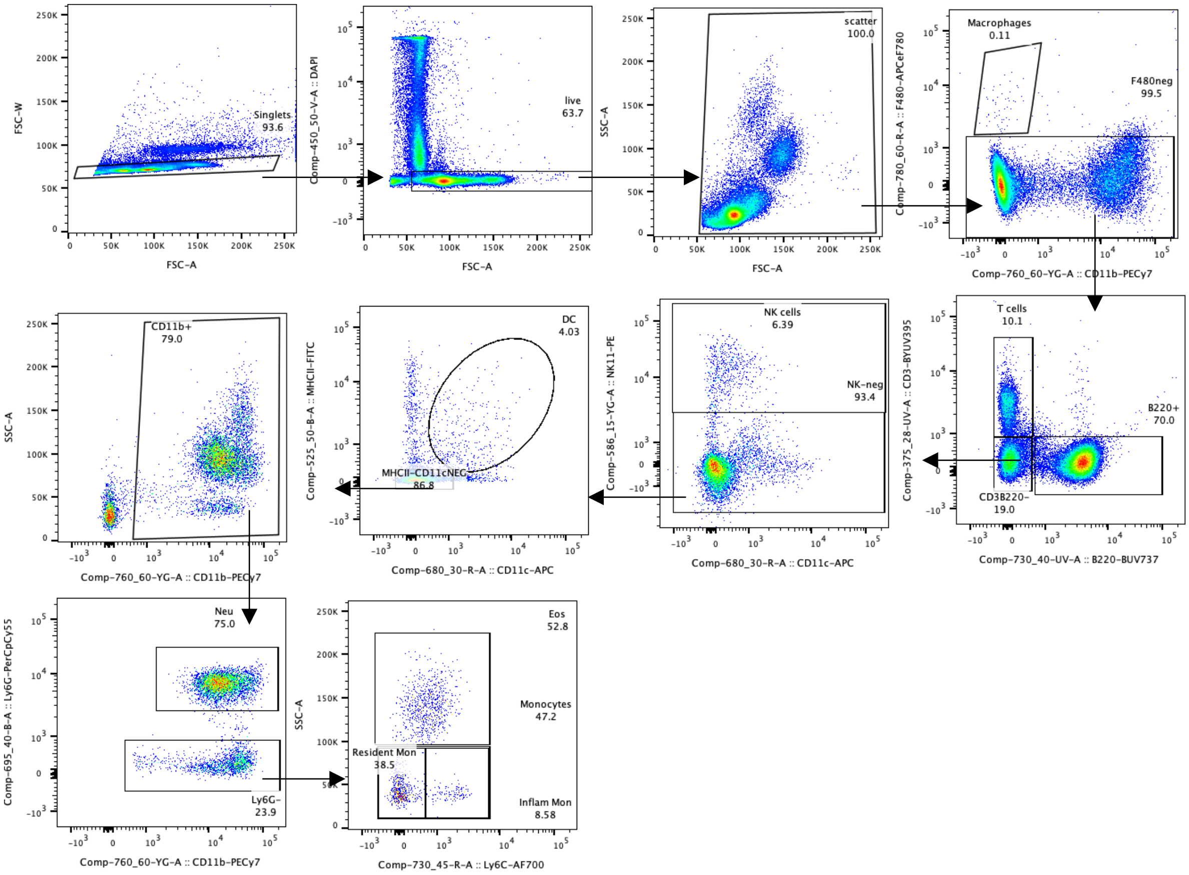


## Gating strategy Figure 4L

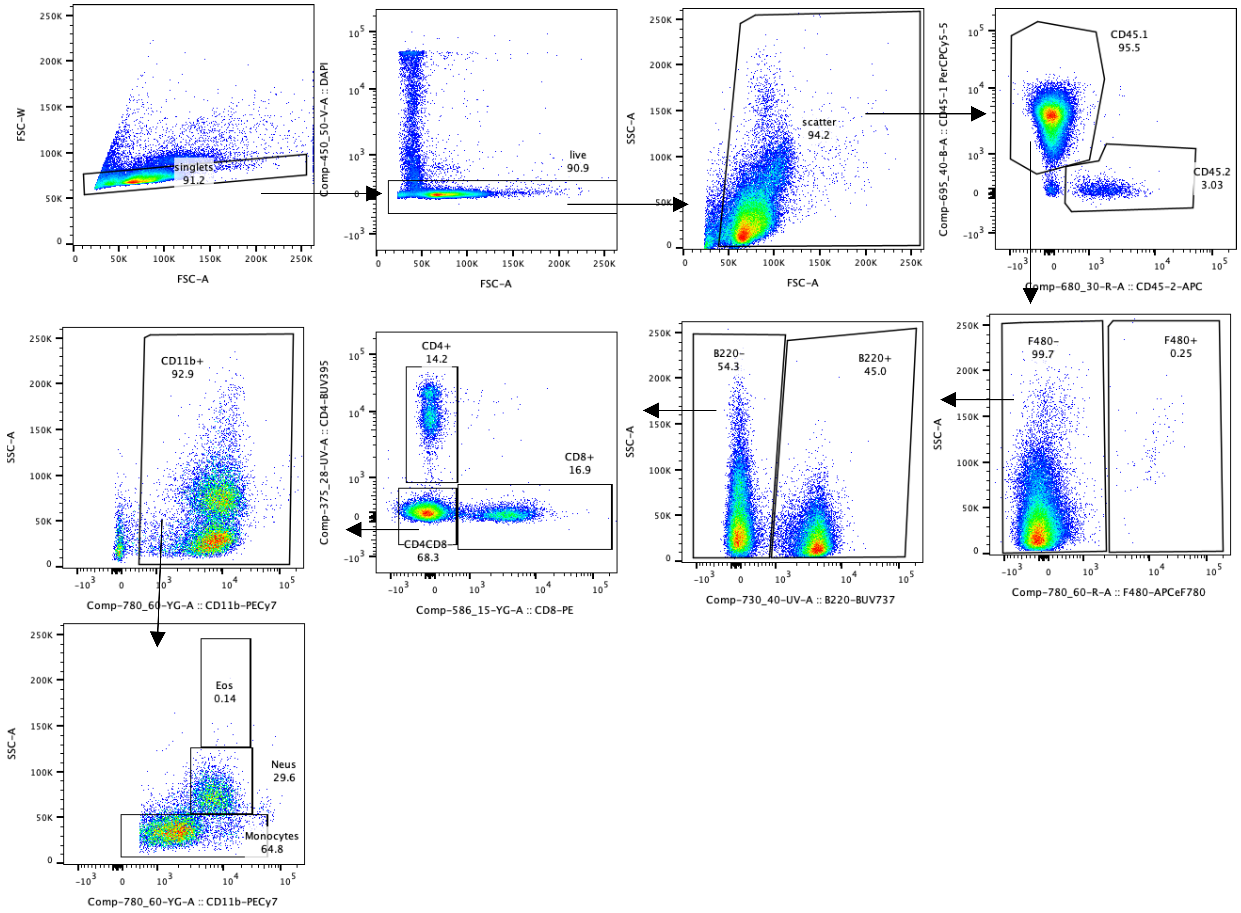

## Gating strategy Figure 5E, 5H

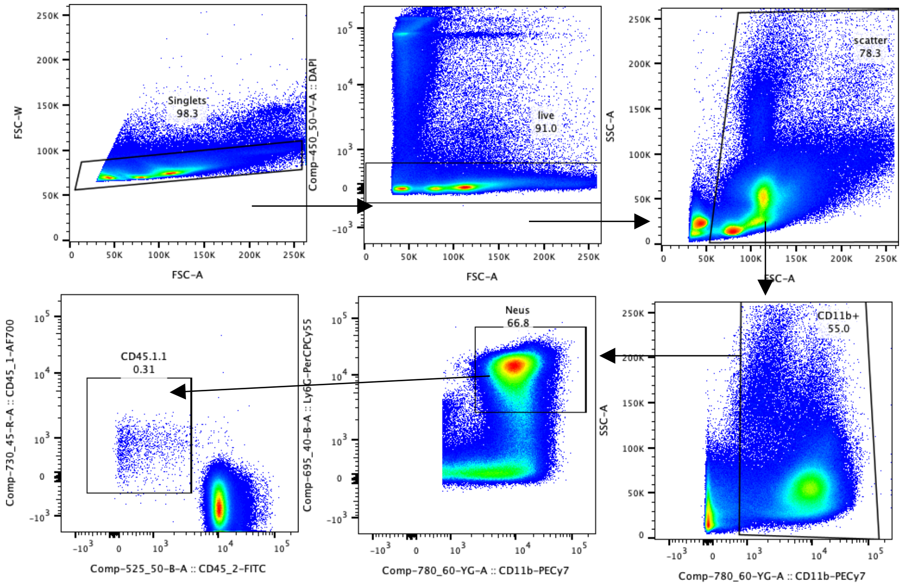


## Gating strategy Supplementary Figure 5G

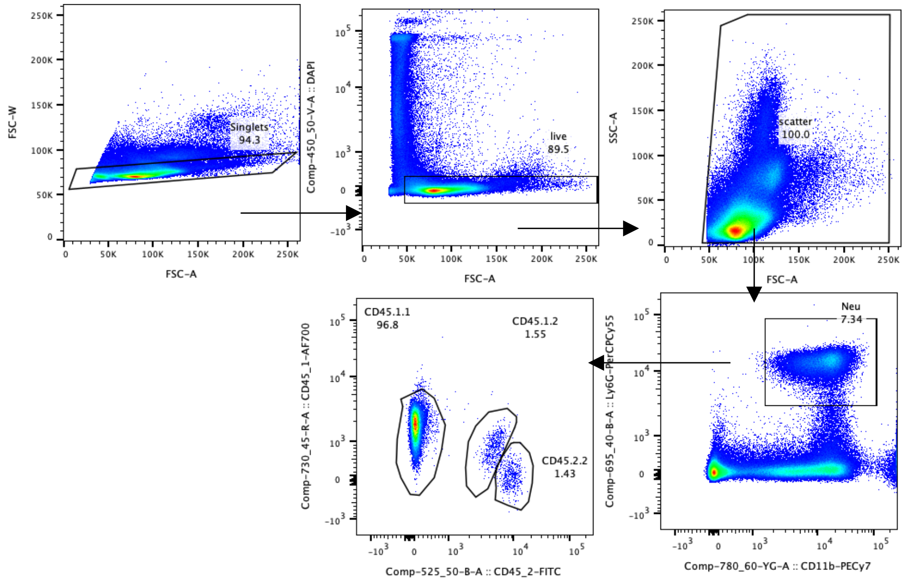


## Gating strategy Figure 7A, Supplementary Figure 7E

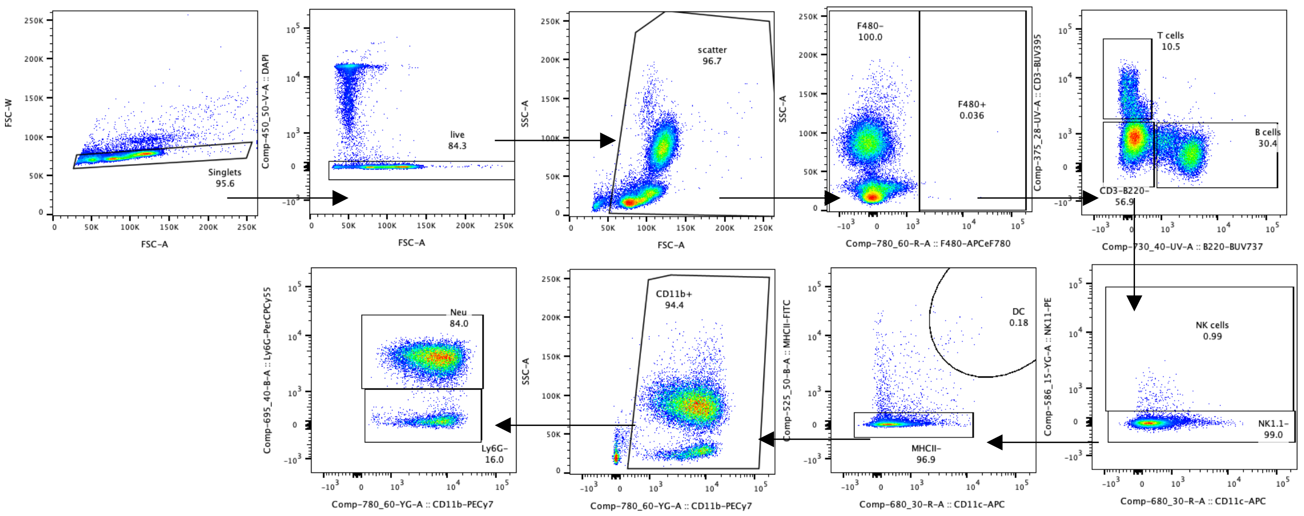

Supplement: Supplementary file 1 — Supplementary Information on gating strategy for FACS. [file 43587_2024_635_MOESM1_ESM.pdf]
