# Supplementary material for: A mild increase in nutrient signaling to mTORC1 in mice leads to parenchymal damage, myeloid inflammation and shortened lifespan
Source: Nat Aging. 2024 Jun 7;4(8):1102–20. doi: 10.1038/s43587-024-00635-x (PMC11333293; doi:10.1038/s43587-024-00635-x)
Supplement: Supplementary file 2 — Reporting Summary [file 43587_2024_635_MOESM2_ESM.pdf]

nature portfolio

Corresponding author(s): Efeyan A; Ortega-Molina A.

Last updated by author(s): Apr 9, 2024

Reporting Summary

Nature Portfolio wishes to improve the reproducibility of the work that we publish. This form provides structure for consistency and transparency in reporting. For further information on Nature Portfolio policies, see our Editorial Policies and the Editorial Policy Checklist.

Statistics

For all statistical analyses, confirm that the following items are present in the figure legend, table legend, main text, or Methods section.

n/a	Confirmed	
☐	☒	The exact sample size (*n*) for each experimental group/condition, given as a discrete number and unit of measurement
☐	☒	A statement on whether measurements were taken from distinct samples or whether the same sample was measured repeatedly
☐	☒	The statistical test(s) used AND whether they are one- or two-sided<br>*Only common tests should be described solely by name; describe more complex techniques in the Methods section.*
☒	☐	A description of all covariates tested
☐	☒	A description of any assumptions or corrections, such as tests of normality and adjustment for multiple comparisons
☐	☒	A full description of the statistical parameters including central tendency (e.g. means) or other basic estimates (e.g. regression coefficient) AND variation (e.g. standard deviation) or associated estimates of uncertainty (e.g. confidence intervals)
☐	☒	For null hypothesis testing, the test statistic (e.g. *F*, *t*, *r*) with confidence intervals, effect sizes, degrees of freedom and *P* value noted<br>*Give P values as exact values whenever suitable.*
☒	☐	For Bayesian analysis, information on the choice of priors and Markov chain Monte Carlo settings
☒	☐	For hierarchical and complex designs, identification of the appropriate level for tests and full reporting of outcomes
☒	☐	Estimates of effect sizes (e.g. Cohen's *d*, Pearson's *r*), indicating how they were calculated

Our web collection on statistics for biologists contains articles on many of the points above.

Software and code

Policy information about availability of computer code

Data collection	Odyssey Infrared Imaging System. Application software version 3.0.30. LI-COR Biosciences, NDP.view2 software<br>AxioScan Z1, Zeiss<br>BD FACSDiva software (BD Biosciences)
Data analysis	GraphPad Prism v9 ; https://www.socscistatistics.com/; AxioVision 4.6 software package;FIJI- ImageJ v1.53, FlowJo v9.8.1 and v10

For manuscripts utilizing custom algorithms or software that are central to the research but not yet described in published literature, software must be made available to editors and reviewers. We strongly encourage code deposition in a community repository (e.g. GitHub). See the Nature Portfolio guidelines for submitting code & software for further information.

Data

Policy information about availability of data

All manuscripts must include a data availability statement. This statement should provide the following information, where applicable:

- Accession codes, unique identifiers, or web links for publicly available datasets
- A description of any restrictions on data availability
- For clinical datasets or third party data, please ensure that the statement adheres to our policy

The transcriptomics data generated in this study have been deposited to the GEO database under the following accession codes:
GSE255148 Gene expression profiling in old and young kidneys from wild type mice (CNIO cohort)

GSE221283: Gene expression profiling in old kidneys from RagC S74N/+ and RagC +/+ mice
GSE221284: Gene expression profiling in young kidneys from RagC S74N/+ and RagC +/+ mice
GSE221285: Gene expression profiling in young neutrophils from RagC S74N/+ and RagC +/+ mice
GSE221286: Gene expression profiling in young livers from RagC S74N/+ and RagC +/+ mice
GSE255864: Gene expression changes in mouse kidney between 4 and 24-26 months of age (NIA cohort)

Source data file is provide with this paper

Research involving human participants, their data, or biological material

Policy information about studies with human participants or human data. See also policy information about sex, gender (identity/presentation), and sexual orientation and race, ethnicity and racism.

Reporting on sex and gender

The analysis of human transcriptional data on Figure 3 and Extended Data Figure 3 were extracted from a cited publication by co-authors of the paper (refernce 51), and as such all relevant information is presented in the published report. As summary the young group includes 3 men (28, 38, 41 years old), 4 women (24, 25, 26, 31 years old) and one case is not reported. Regarding the old group, includes six women (74, 74, 74, 75, 75, 75 years old).

Reporting on race, ethnicity, or other socially relevant groupings

N/A

Population characteristics

The Spanish Centenarian Study Group at RETICEF, began in 2007 as a population-based study of all centenarians living within an area near of Valencia called La Ribera (11th Health Department of the Valencian Community, Spain), which is composed of 29 towns (240.000 inhabitants). Potential subjects were selected from the population data system of the 11th Health Department. We found 31 centenarians of whom 20 met the inclusion criteria.

Recruitment

Randomly recruited 20 older adult of whom 16 met the inclusion criteria and 20 young people of whom 14 fulfilled the inclusion criteria. The inclusion criteria were: to be born within the dates indicated in the study (before 1908 for centenarians, between 1928 and 1938 for octogenarians and between 1968 and 1988 for young individuals), to live in the 11th Health Department for at least the last 6 years and to sign the informed consent. The exclusion criterion was to be terminally ill for any reason (from ref. 51).

Ethics oversight

The Ethics approval certificate for the analysis of data was issued on February 5th 2010 by the Ethics Committee of the Hospital Universitario de la Ribera de Alzira. All patients or their relatives were fully informed of the aims and scope of the research and signed an informed consent. No compensation was received by the participants.

Note that full information on the approval of the study protocol must also be provided in the manuscript.

Field-specific reporting

Please select the one below that is the best fit for your research. If you are not sure, read the appropriate sections before making your selection.

☒ Life sciences ☐ Behavioural & social sciences ☐ Ecological, evolutionary & environmental sciences

For a reference copy of the document with all sections, see nature.com/documents/nr-reporting-summary-flat.pdf

Life sciences study design

All studies must disclose on these points even when the disclosure is negative.

Sample size

We did not estimate the sample size calculation for most studies, as the magnitude of the effect sizes were unknown. As reference, the sample sizes were guided on the basis of similar published studies (22405073, 31579886, 34135321, 34260908, 38499523).
Details on sample size of all experiments are provided in the Methods section and figure legends

Data exclusions

No data were excluded unless as determined by technical problems, such as low RNA quality, low number of cells.

Replication

All attempts of replication (at least twice) under independent conditions were successful

Randomization

Mice were randomly assigned to different treatments/conditions.

Blinding

Mice studies, including aging were performed in a blinded fashion. Investigators were blinded to genotype as mice have numbered IDs but no information on genotypes is available in cage cards. Genotypes were verified at the analysis step.
To diagnose aging features and pathologies, the genotype of the mouse was not disclosed to the pathologist.

Reporting for specific materials, systems and methods

We require information from authors about some types of materials, experimental systems and methods used in many studies. Here, indicate whether each material, system or method listed is relevant to your study. If you are not sure if a list item applies to your research, read the appropriate section before selecting a response.

Materials & experimental systems

n/a	Involved in the study
☐	☒ Antibodies
☒	☐ Eukaryotic cell lines
☒	☐ Palaeontology and archaeology
☐	☒ Animals and other organisms
☒	☐ Clinical data
☒	☐ Dual use research of concern
☒	☐ Plants

Methods

n/a	Involved in the study
☒	☐ ChIP-seq
☐	☒ Flow cytometry
☒	☐ MRI-based neuroimaging

Antibodies

Antibodies used

CD45.1.1-AF700 A20 110724 Biolegend
CD45.2.2-FITC 104 109806 Biolegend
CD11b-PECy7 M1/70 552850 BD Pharmingen™
F4/80-APCeF780 BM8 47-4801-82 eBioscience
NK.1.1-PE PK136 50-5941-U100 TONBO Biosciences
B220-BUV737 RA3-6B2 612838 BD Horizon™
CD3-BUV395 145-2C11 563565 BD Horizon™
MHC Class II (I-A/I-E)-FITC M5/114.15.2 35-5321-U100 TONBO Biosciences
CD11c-APC N418 20-0114-U100 TONBO Biosciences
Ly6G-PerCPCy5.5 1A8 65-1276-U100 TONBO Biosciences
Ly6G-AF700 1A8 56-9668-82 Invitrogen
Ly6G-BUV737 1A8 741813 BD Bioscience
Ly6C-AF700 HK1.4 128024 BioLegend
CXCR4-PE 2B11 12-9991-82 eBioscience
CXCR2-PerCPCy5.5 SA044G4 149308 BioLegend
CD45-eF506 30-F11 69-0451-82 Invitrogen
CD62L-SB702 MEL-14 67-0621-82 ThermoFisher

p21 (F-5) Mouse sc-6246 Santa Cruz Biotech
phospho-S6 235/236 Rabbit 2211 Cell Signaling Technology
Phospho-T389-S6K1; Rabbit, 9234 Cell Signaling Technology
S6K1, Rabbit, 2708 Cell Signaling Technology
Tfeb Rabbit A303-673A Bethyl Lab
Tfe3 Rabbit HPA023881 Sigma-Aldrich
b-actin Mouse A5441 Sigma-Aldrich
Vcam1 Rabbit 39036 Cell Signaling Technology
Gapdh Mouse G8795 Sigma-Aldrich
H3.3 (clone RM190) Rabbit Monoclonal Antibody 31-1058-00 RevMab Biosciences
Phospho-Histone H2A.X (Ser139) (20E3) Rabbit 9718 Cell Signaling Technology
IRDYE 680RD goat α-mouse Goat 926-68070 LICOR
IRDYE 800CW goat α-rabbit Goat 926-32211 LICOR

anti-p21 (clone 291HUGO; antibody made by CNIO Monoclonal Antibodies Core Unit),also commercialized by Abcam (#AB107099).
rabbit polyclonal Myeloperoxidase (MPO, DAKO, #A0398),
rabbit polyclonal CD45 (clone D3F8Q, Cell Signaling Technology, #70257),
rabbit monoclonal Vcam1 (clone D2T4N, Cell Signaling Technology, #32653)

Goat anti-Rabbit IgG (H+L) Cross-Adsorbed Secondary Antibody, Alexa Fluor™ 488 (Invitrogen #A-11008)

anti-Gr1 antibody (BioXcell, clone RB6-8C5, #BE0075)
isotype control (BioXcell, clone LTF-2, #BE0090)
anti-Vcam1 antibody (Bioxcell, clone M/K-2.7, #BE0027-25MG)

Validation

CD45.1.1-AF700 A20 110724 Biolegend. Used for Flow Citometry. Validated by the company and by users (cited 49 times)
CD45.2.2-FITC 104 109806 Biolegend . Used for Flow Citometry. Validated by the company and by users (cited 103 times)
CD11b-PECy7 M1/70 552850 BD Pharmingen™ . Used for Flow Citometry. Validated by the company and by users (cited 10 times)
F4/80-APCeF780 BM8 47-4801-82 eBioscience . Used for Flow Citometry. Validated by the company and by users (cited 51 times)
NK.1.1-PE PK136 50-5941-U100 TONBO Biosciences. Used for Flow Citometry. Validated by the company
B220-BUV737 RA3-6B2 612838 BD Horizon™ . Used for Flow Citometry. Validated by the company and by users (cited 15 times)
CD3-BUV395 145-2C11 563565 BD Horizon™ . Used for Flow Citometry. Validated by the company and by users (cited 14 times)
MHC Class II (I-A/I-E)-FITC M5/114.15.2 35-5321-U100 TONBO Biosciences. Used for Flow Citometry. Validated by the company
CD11c-APC N418 20-0114-U100 TONBO Biosciences. Used for Flow Citometry. Validated by the company
Ly6G-PerCPCy5.5 1A8 65-1276-U100 TONBO Biosciences. Used for Flow Citometry. Validated by the company
Ly6G-AF700 1A8 56-9668-82 Invitrogen . Used for Flow Citometry. Validated by the company and by users (cited 17 times)
Ly6G-BUV737 1A8 741813 BD Bioscience . Used for Flow Citometry. Validated by the company and by users (cited 3 times)
Ly6C-AF700 HK1.4 128024 BioLegend. Used for Flow Citometry. Validated by the company and by users (cited 35 times)

CXCR4-PE 2B11 12-9991-82 eBioscience. Used for Flow Citometry. Validated by the company and by users (cited 22 times)
CXCR2-PerCPCy5.5 SA044G4 149308 BioLegend. Used for Flow Citometry. Validated by the company and by users (cited 5times)
CD45-eF506 30-F11 69-0451-82 Invitrogen . Used for Flow Citometry. Validated by the company and by users (cited 228 times)
CD62L-SB702 MEL-14 67-0621-82 ThermoFisher. Used for Flow Citometry. Validated by the company and by users (cited 24 times)

p21 (F-5) Mouse sc-6246 Santa Cruz Biotech. Used for WB in mouse tissues. Validated by the company and by users (cited 1811 times)
phospho-S6 235/236 Rabbit 2211 Cell Signaling Technology. Used for WB in mouse tissues. Validated by the company and by users (cited 1470 times)
Phospho-T389-S6K1; Rabbit, 9234 Cell Signaling Technology. Used for WB in MEFs Validated by the company and by users (cited 857 times)
S6K1, Rabbit, 2708 Cell Signaling Technology. Used for WB in MEFs. Validated by the company and by users (cited 801 times)
Tfeb Rabbit A303-673A Bethyl Lab. Used for WB in mouse tissues and MEFs and for immunohistochemistry. Validated in the manuscript
Tfe3 Rabbit HPA023881 Sigma-Aldrich. Used for WB in mouse tissues. Validated by the company and by users (cited 88 times)
b-actin Mouse A5441 Sigma-Aldrich. Used for WB in mouse tissues and mouse cells. Validated by the company and by users (cited 10103 times)
Vcam1 Rabbit 39036 Cell Signaling Technology. Used for WB in mouse tissues. Validated by the company and by users (cited 27 times)
Gapdh Mouse G8795 Sigma-Aldrich. Used for WB in mouse tissues. Validated by the company and by users (cited 1242 times)
H3.3 (clone RM190) Rabbit Monoclonal antibody. 31-1058-00 RevMab Biosciences. Used for WB in mouse tissues. Validated by the company and by users (cited 1 time)
Phospho-Histone H2A.X (Ser139) (20E3) Rabbit 9718 Cell Signaling Technology. Validated by the company and by users (cited 641 times)
IRDYE 680RD goat α-mouse Goat 926-68070 LICOR. Secondary antibody. Used for WB in mouse tissues and cells. Validated by the company
IRDYE 800CW goat α-rabbit Goat 926-32211 LICOR. Secondary antibody. Used for WB in mouse tissues and cells. Validated by the company

anti-p21 (clone 291HUGO; CNIO Monoclonal Antibodies Core Unit, also commercialized by ABCAM# AB107099). Used for immunohistochemistry. Validated by the unit (cited more that 30 times)
rabbit polyclonal Myeloperoxidase (MPO, DAKO, #A0398). Used for immunohistochemistry. Validated by users (cited 411 times)
rabbit polyclonal CD45 (clone D3F8Q, Cell Signaling Technology, #70257). Used for immunohistochemistry. Validated by users (cited 49 times)
rabbit monoclonal Vcam1 (clone D2T4N, Cell Signaling Technology, #32653) . Used for immunohistochemistry. Validated by users (cited 9 times)

Goat anti-Rabbit IgG (H+L) Cross-Adsorbed Secondary Antibody, Alexa Fluor™ 488 (Invitrogen #A-11008). Used for immunofluorescence. Validated by users (cited 8928 times)

anti-Gr1 antibody (BioXcell, clone RB6-8C5, #BE0075). Used for for in vivo depletion of Gr-1+ myeloid cells. Validated for in vivo depletion of Gr-1+ myeloid cells (cited 14 times)
isotype control (BioXcell, clone LTF-2, #BE0090). Used as isotype control. Validated by the company and by the users (cited 15 times)
anti-Vcam1 antibody (Bioxcell, clone M/K-2.7, #BE0027-25MG). Used for or in vivo VCAM-1 neutralization . Validated for in vivo VCAM-1 neutralization (cited 7 times)

Animals and other research organisms

Policy information about studies involving animals; ARRIVE guidelines recommended for reporting animal research, and Sex and Gender in Research

Laboratory animals

Mus musculus, C57BL/6, males and females, from E13.5 to 2 years old. Specific strains:RagC +/+, RagC S74N/+, RagC S74C/+, RagC T89N/+, IghmμMT (JAX stock no. 002288)
Mice at CNIO were housed under specific pathogen free conditions at 22ºC and 55±10% humidity with 12h dark/light cycles (light cycle from 8AM to 8PM). Mice were fed with standard chow diet (Harlan Teklad #2018S/2018SC). All animal procedures carried out at the CNIO were performed according to protocols approved by the CNIO-ISCII Ethics Committee for Research and Animal Welfare (CEIyBA) and the Autonomous Community of Madrid (CAM). Protocol numbers PROEX285/15, PROEX15/18 and PROEX225.7/22.

Mice at NIA (C57BL/6) were housed in rooms maintained at 22.2 ± 1ºC and 30%–70% humidity. Routine tests were performed to ensure that mice were pathogen-free and sentinel cages were maintained and tested according to American Association for Accreditation of Laboratory Animal Care criteria. This study was approved by the Animal Care and Use Committee of the NIA in Baltimore, MD (protocol number 277-TGB-2022)

Wild animals

not used

Reporting on sex

the use of males or females is clearly reported in the figure legend

Field-collected samples

no field-collected samples

Ethics oversight

All animal procedures carried out at the CNIO were performed according to protocols approved by the CNIO-ISCII Ethics Committee for Research and Animal Welfare (CEIyBA) and the Autonomous Community of Madrid (CAM). Protocol numbers PROEX285/15, PROEX15/18 and PROEX225.7/22.
All animal procedures carried out at the NIA were performed according to protocols approved by the Animal Care and Use

Committee of the NIA in Baltimore, MD (protocol number 277-TGB-2022)

Note that full information on the approval of the study protocol must also be provided in the manuscript.

Plants

Seed stocks	*Report on the source of all seed stocks or other plant material used. If applicable, state the seed stock centre and catalogue number. If plant specimens were collected from the field, describe the collection location, date and sampling procedures.*
Novel plant genotypes	*Describe the methods by which all novel plant genotypes were produced. This includes those generated by transgenic approaches, gene editing, chemical/radiation-based mutagenesis and hybridization. For transgenic lines, describe the transformation method, the number of independent lines analyzed and the generation upon which experiments were performed. For gene-edited lines, describe the editor used, the endogenous sequence targeted for editing, the targeting guide RNA sequence (if applicable) and how the editor was applied.*
Authentication	*Describe any authentication procedures for each seed stock used or novel genotype generated. Describe any experiments used to assess the effect of a mutation and, where applicable, how potential secondary effects (e.g. second site T-DNA insertions, mosiacism, off-target gene editing) were examined.*

Flow Cytometry

Plots

Confirm that:

☒ The axis labels state the marker and fluorochrome used (e.g. CD4-FITC).

☒ The axis scales are clearly visible. Include numbers along axes only for bottom left plot of group (a 'group' is an analysis of identical markers).

☒ All plots are contour plots with outliers or pseudocolor plots.

☒ A numerical value for number of cells or percentage (with statistics) is provided.

Methodology

Sample preparation	Mononuclear cell pools (from spleen or Bone marrow) were isolated from mouse at the indicated times. Cells were separated by crushing the spleens through a 70 micrometer mesh (Corning) in ice-cold PBS +0.1% BSA +3mM EDTA, and red blood cells were lysed using Erythrocyte Lysis Buffer (Qiagen, #79217). Cell staining was performed on ice in PBS +0.1% BSA + 3mM EDTA
Instrument	BD Canto II, LSR-Fortessa, BD FACSAria Ilu, InFlux (Cytopeia-Becton Dickinson
Software	BD FACSDiva software (BD Biosciences). FlowJo software (v 9.8.1 and v.10; TreeStar)
Cell population abundance	sorted neutrophils were >90% (CD45+, CD11b+, Ly6G+)
Gating strategy	Macrophages were identified as F4/80+/SSChi.<br>NK cells were identified as F4/80-B220-/CD3-/NK.1.1+.<br>Dendritic cells were identified as F4/80-B220-/CD3-/NK.1.1-/MHCII+/CD11c+.<br>Neutrophils were identified as F4/80-/B220- /CD3-/NK.1.1-/MHCII-/CD11c-/CD11b+/Ly6G+.<br>Monocytes were identified as F4/80-/B220-/CD3-/NK.1.1-/MHCII-/CD11c-/CD11b+/Ly6G-/Ly6Clow or high.<br>Eosinophils were identified as F/480-/B220-/CD3-/NK.1.1-/MHCII-/CD11c-/CD11b+/Ly6G-/Ly6Cmed.<br>Aged neutrophils were identified as CD11b+/Ly6G+/CD62L-/CXCR4+.<br>Fresh neutrophils were identified as CD11b+/Ly6G+/CD62L+/CXCR2+.

☒ Tick this box to confirm that a figure exemplifying the gating strategy is provided in the Supplementary Information.
